# Negative observational learning might play a limited role in the cultural evolution of technology

**DOI:** 10.1038/s41598-022-05031-2

**Published:** 2022-01-19

**Authors:** Yo Nakawake, Yutaka Kobayashi

**Affiliations:** 1 School of Economics and Management, Kochi University of Technology, Kochi, 780-8515 Japan; 2 School of Anthropology and Museum Ethnography, University of Oxford, Oxford, OX2 6PE UK

**Keywords:** Evolution, Cultural evolution

## Abstract

Theoretical and empirical studies of the cultural evolution in technology have often focused on positive observational learning, i.e., copying a successful individual. However, negative observational learning, i.e., avoiding negative or bad exemplar behavior, is ubiquitous in humans and other animals. In this paper, we experimentally investigate whether observing negative examples can assist in tool making in the virtual arrowhead task, which has been widely applied to test the theory of cultural evolution in the technological domain. We set three conditions that differ in the kinds of social learning available to participants: (1) positive observational learning, (2) negative observational learning, and (3) pure asocial learning. The results of the positive observational and pure asocial learning conditions replicated previous studies; i.e., participants in the positive observational learning condition outperformed those in the asocial learning condition. In contrast, opportunities to observe negative examples did not increase the performance compared to pure asocial learning. Computer simulations in the same setting showed that the presence of negative exemplars is in theory beneficial to participants, providing additional pieces of information on the relationship between arrowhead designs and their performance scores. These findings together suggest that negative observational learning might play only a limited role in the cultural evolution of technologies possibly due to a cognitive bias in humans toward copying.

## Introduction

According to the prevailing view, high-fidelity transmission of information across generations is essential to cumulative cultural evolution, allowing new generations to build upon the advances established by preceding generations^1,2^. Understandably, recent studies in developmental and comparative cognition have sought cognitive capacities that enable faithful informational transmission uniquely developed in humans^3–6^. Plausible candidates are overimitation^7–9^, mentalizing^10^, production and understanding of ostensive signals^11^, or shared intentionality^12^. The Vygotskian intelligence^13^ or cultural brain hypothesis^14^ claims that those social capacities enabling high-fidelity transmission, rather than individual cognitive intelligence (e.g., rationality or causal reasoning), are the key to understanding human cultural complexity. Thus, synonymous usage of social learning and copying (i.e., transmission of behavior through social learning) is deeply entrenched in the field of cultural evolution^15^, where research attention has mostly been paid to the context^16^ and contents^17–19^ of copying, although the term ‘social learning’ itself has a much broader meaning^15^.

Some researchers, however, suggested that faithful transmission^20–22^ or even copying itself^23^ might not be a prerequisite for cumulative cultural evolution. For example, a transmission chain experiment of visual patterns showed that cumulative cultural evolution can occur without copying (i.e., more structured visual patterns emerged through transmissions); in this experiment, however, each participant was rewarded when their behavior was dissimilar to that of the previous participant in the same transmission chain^23^. Conversely, in the cultural evolution of technologies, which is the focus of the present study, the performance of an artifact should be determined by its degree of adaptation to the environment rather than by similarity or dissimilarity to the artifacts of exemplars. Therefore, there is room to discuss whether cumulative cultural evolution without copying is also possible for technological traits. In theory, the presence of unsuccessful neighbors should be a useful source of social learning, allowing observers to avoid learning the same mistakes on their own through trial and error. Interestingly, however, the abovementioned transmission chain experiment suggested that human children may have a stronger bias toward copying than do baboons^23^. Such a bias, if manifested in the transmission of artifact designs, might impede humans from utilizing social information obtained through the observation of low-performance exemplars in a rational and efficient manner.

Social learning from positive or successful outcomes is well documented in theoretical and empirical studies under the names of *copy-successful-individuals*^17,24^*, prestige-biased*^25–27^ or *payoff-biased*^28^ learning. However, learning based on a negative or unsuccessful outcome is scarcely studied in the context of cumulative cultural evolution, especially in terms of cultural evolution in the technological domain (except one theoretical study^29^).

Note that several types of learning mechanisms related to negative experiences of conspecific individuals are widely observed in various species and contexts, although these mechanisms are very different from each other and should not be lumped together under the heading of ‘negative observational learning’. Here we briefly present three of these types. (1) One type, found widely in animals, is fear learning through witnessing unfortunate events that other individuals happen to experience: vicariously learning not to do a certain behavior or not to approach a certain target through negative conditioning^30^. Examples range from avoidance of toxic food in Norway rats (*Rattus norvegicus*)^31^ to predator recognition in woodfrog tadpoles (*Rana sylvatica*) acquired through predation of conspecifics^32^. (2) The second type is learning to avoid danger through signals related to others’ negative experiences. For example, animals can learn to avoid the odor of predators by observing conspecifics’ fear responses to the odor^32^. Likewise, oral transmission allows humans to transmit the avoidance of cues related to predators^33,34^. Note that in this type of learning learners do not directly observe negative experiences of others unlike in the first type. (3) The third type, which is the most relevant to the present study, is learning through witnessing failed attempts by others to achieve a certain specific goal. A previous study of the three-armed bandit task (finding treasure by choosing from three options) showed that human children (3–6 years old) could beneficially exploit negative social information (i.e., failure of others) when it is combined with positive social information^35^. Further, an experimental study showed that 3-year-old children were more likely to learn correct tool-use when negative and positive social information were given together than when only positive social information was given; the performance did not significantly increase compared with chance level when only negative social information was given^36^. Those studies suggested that from early childhood, humans can already exploit negative outcomes of others’ actions to get their own desirable outcomes.

While those empirical studies showed potential benefits of acquiring negative social information, its role in the cumulative cultural evolution of technology remains unclear. Nakahashi’s model^29^ showed that negative observational learning could in theory foster the speed of cumulative cultural evolution of technology. While usual models of cultural evolution assume that individuals learn socially from the most successful members of the group^37,38^, Nakahashi’s model deals explicitly with the process through which individuals select their role models^29^. In this model, the probability that an individual eventually reaches a good exemplar increases with the variance in performance among exemplars; that is, the presence of low-performance exemplars helps guide novices to successful exemplars^29^. Although the observation of low-performance individuals accelerates cumulative cultural evolution, the *copy-successful-individuals* mechanism still plays a pivotal role in his model.

In this paper, we investigate whether negative observational learning contributes to the technological performance of individuals in a laboratory experiment. The framework we use is the virtual arrowhead task, which is a computer-based task originally developed by Alex Mesoudi^17,39–42^. In this task, each participant designs a virtual arrowhead on a computer display, where the performance of each design is determined by an exogenously defined fitness landscape and an additional noise factor. The participants can access only the fitness outcomes of their artifacts but not directly the shape of the underlying landscape itself. This framework was originally introduced to explain the cultural variability of archaeological artifacts in the Great Basin (Nevada and California in the US) and was subsequently applied to the investigation of the social learning strategies of individuals^17^, their cultural differences^42^, or the role of inductive bias in cultural evolution^43^. Using this framework, a series of experiments showed that the participants were highly dependent on social information and tended to copy the most successful individual^17,39,41,42^. In our experiment, unlike in previous experiments, participants provided with social information could access either only positive information (i.e., designs better than theirs) or only negative information (i.e., designs worse than theirs), depending on condition. We also set an asocial condition in which participants could not access any social information as the baseline case. This setting enabled us to test whether the presence of negative information could increase performance compared to the asocial condition and, if so, whether it would be as effective as positive information.

Prior to the experiment, we hypothesized that participants in the (i) positive and (ii) negative social learning conditions would both outperform those in the asocial learning condition. We tested hypothesis (i) about positive social learning to confirm the consistency with the results of previous studies. We were more interested to test hypothesis (ii) about negative social learning. This hypothesis is rational because participants in the negative social learning condition experience additional useful information that is not available to those in the asocial learning condition. This rationale behind the second hypothesis was indeed confirmed through computer simulations (see “Agent-based computer simulation” section): the simulations show that rational players should be able to exploit the opportunities of negative social learning to perform better than pure asocial learners. However, real humans might not be as rational as agents in the simulations, and hence the second hypothesis was still worth testing through the experiment.

## Results

**Figure 1:**
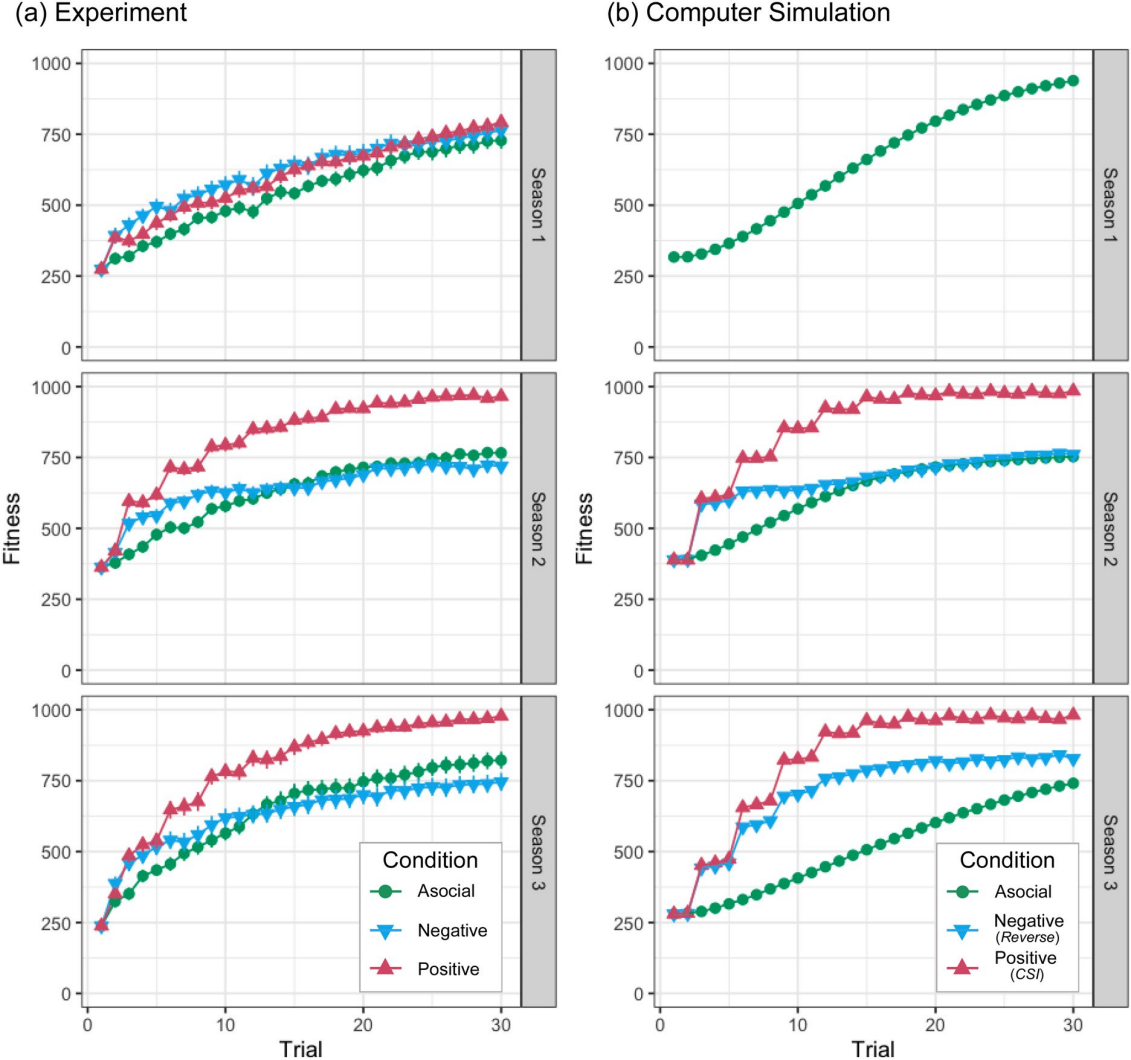
(**a**) Average fitness (performance) of participants’ arrowheads (excluding perception error) against trials. Error bars show standard errors. (**b**) Corresponding results of the computer simulation with the *CSI* and *Reverse* social learning algorithms. The rows correspond to seasons both in (**a,b**).

**Figure 2:**
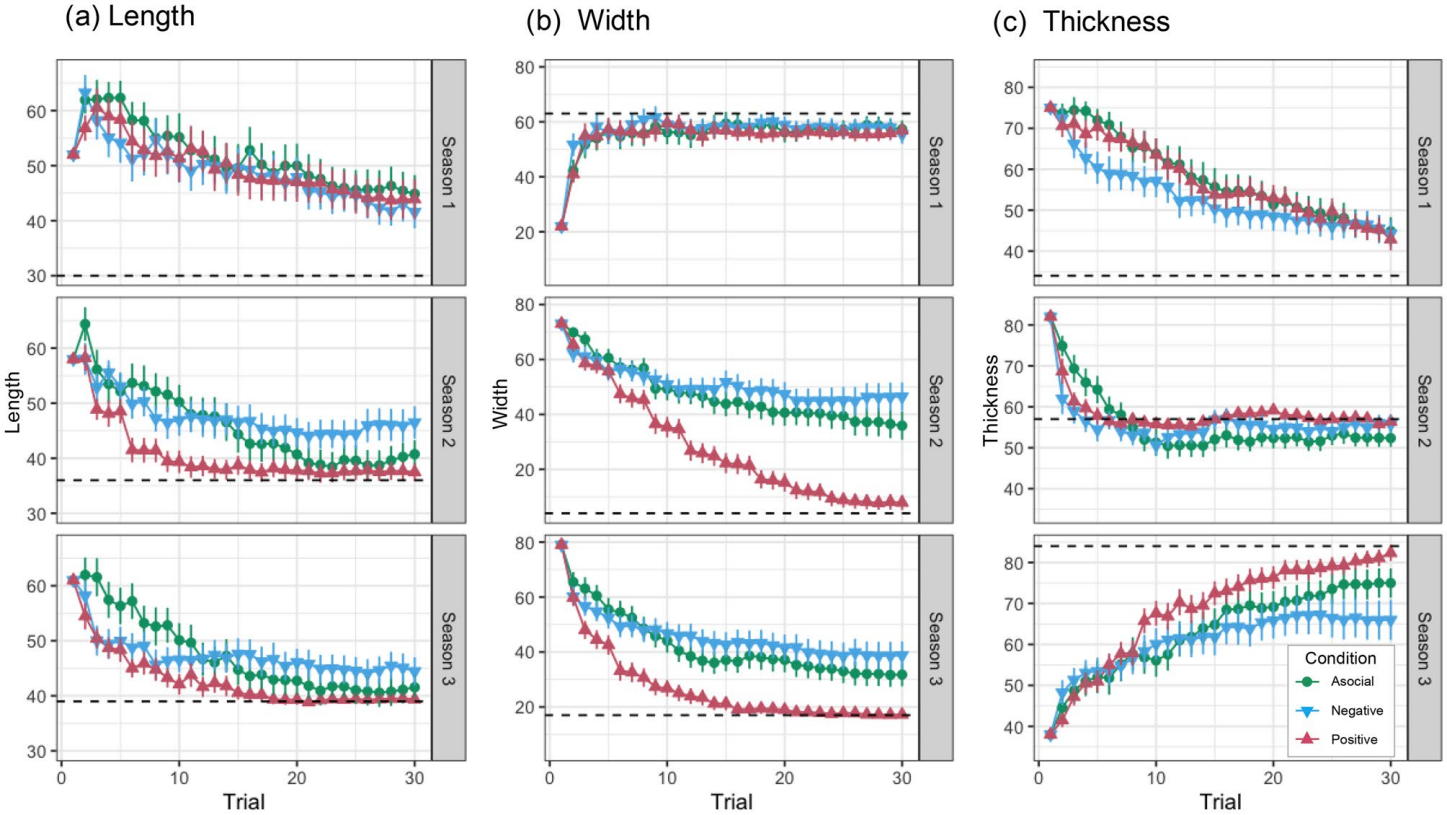
Shifts in the values of length (**a**), width (**b**), and thickness (**c**) across 30 trials. The dotted lines show the optimal value (global optimum) for each attribute. Error bars show standard errors.

The mean score (average performance or fitness) of participants’ arrowheads in each condition is shown in Fig. 1, and the shifts in the values of length, width, and thickness are shown in Fig. 2. Inspection of Fig. 1 revealed that participants in the positive condition performed much better than those in the other two conditions in Seasons 2 and 3. In those seasons, the mean score in the positive condition was consistently higher than that in the asocial condition shortly after the first social learning opportunity and almost reached the maximum (1000 cal) in later trials. While participants in the negative condition performed better than those in the *asocial* condition in the first few trials, the latter soon caught up with, and eventually outstripped, the former.

Following the method of the previous study^17^, we performed *t*-tests to compare each of the two social learning conditions with the asocial condition in terms of the overall performance (i.e., the mean score over the 30 trials). Given that two comparisons were made (i.e., positive vs. asocial; negative vs. asocial) for each of three seasons, the significance criteria were adjusted by the Bonferroni correction method (*α*_*Bonferroni*_ = 0.0083; divided by six, two comparisons for each of three seasons). Participants in the positive condition did not perform significantly better than those in the asocial learning condition in Season 1 (*M*_*positive*_ = 599.9, *SD* = 126.2, *M*_*asocial*_ = 543.4, *SD* = 120.9, *t*_77.6_ = 2.05, *p* = 0.044, *d* = 0.46) but performed significantly better in Season 2 (*M*_*positive*_ = 822.32, *SD* = 87.0, *M*_*asocial*_ = 625.88, *SD* = 97.4, *t*_77.02_ = 9.51, *p* < 0.001, *d* = 2.13) and in Season 3 (*M*_*positive*_ = 796.9, *SD* = 116.2, *M*_*asocial*_ = 639.0, *SD* = 146.8, *t*_74.10_ = 5.33, *p* < 0.001, *d* = 1.19). Participants in the negative condition significantly outperformed those in the asocial condition in none of the three seasons: Season 1 (*M*_*negative*_ = 613.3, *SD* = 162.9, *M*_*asocial*_ = 543.4, *SD* = 120.9, *t*_71.96_ = 2.18, *p* = 0.033, *d* = 0.49), Season 2 (*M*_*negative*_ = 638.2, *SD* = 111.3, *M*_*asocial*_ = 625.88, *SD* = 97.4, *t*_76.67_ = 0.53, *p* = 0.601, *d* = 0.12) and Season 3 (*M*_*negative*_ = 626.9, *SD* = 176.4, *M*_*asocial*_ = 639.0, *SD* = 146.8, *t*_75.51_ =  − 0.33, *p* = 0.740, *d* =  −  0.08).

**Figure 3:**
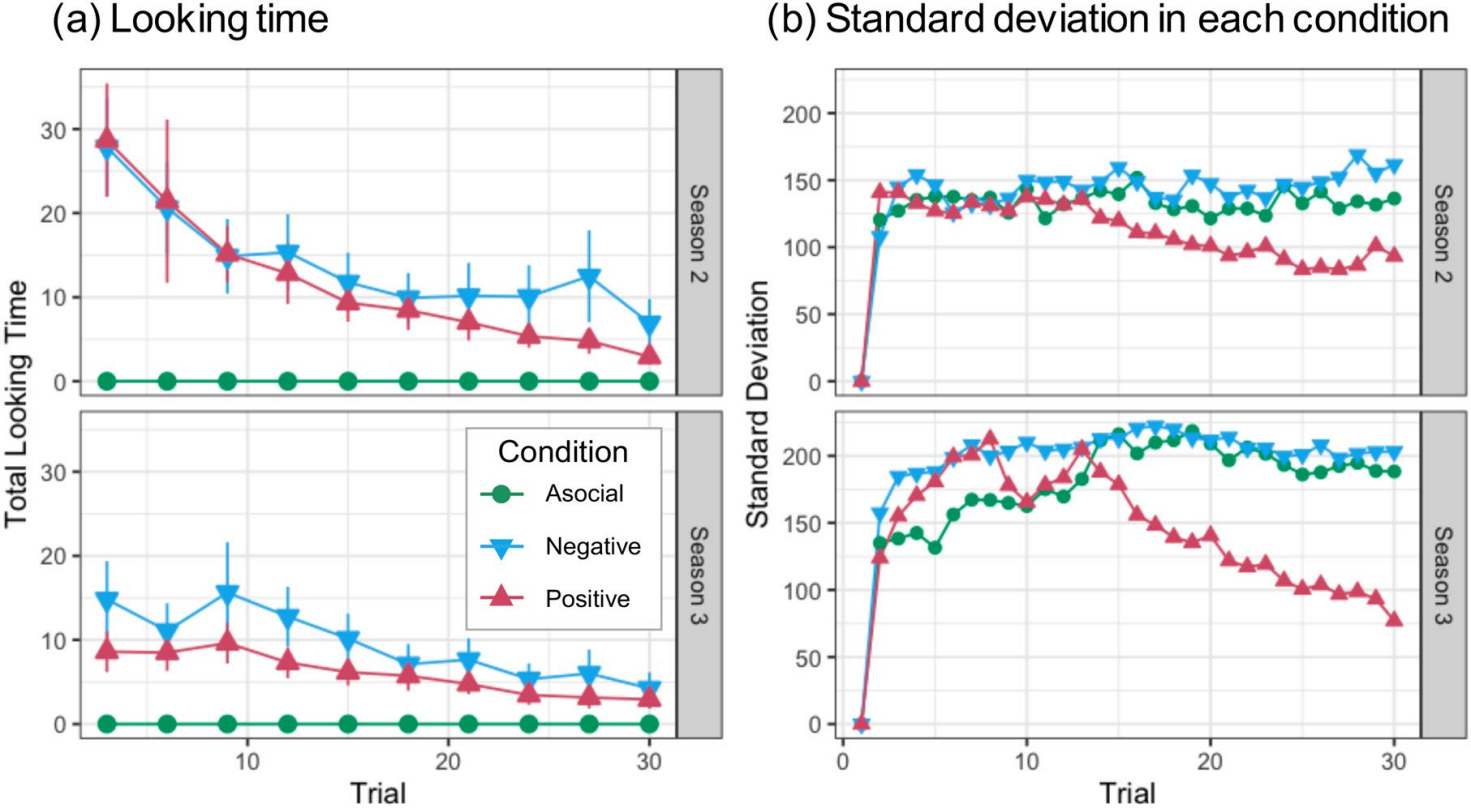
(**a**) Average total looking time (seconds) of social information. Error bars represent standard errors. (**b**) Standard deviation in fitness against trials.

Thus, negative observational learning did not yield appreciable benefits to participants, even though the failure of others should in theory convey useful information. One might suspect that participants in the negative condition considered the social information useless and hence ignored it. However, this is unlikely for at least two reasons. First, the postquestionnaire showed that participants in the negative condition rated social information on average as slightly more useful (*M* = 4.7, *SD* = 1.75) than 4, the neutral middle point (*t*_39_ = 2.45, *p* = 0.019, *d* = 0.39); however, the rating was not as high as in the positive condition, where 80% of participants (32/40) gave the maximum rating of 7 (*M* = 6.5, *SD* = 1.22). Second, the looking time and frequency of social information suggest that negative social information was exploited as much as positive social information was. Figure 3a plots the mean looking time of social information (summed over the four hunters and averaged over participants) against trials. As the figure shows, participants spent as long a time browsing negative information as browsing positive information. Similarly, the frequency of mouse clicks for browsing social information was comparable between the two social learning conditions (see Supplementary Material). In summary, participants in the negative condition might not consider social information very useful but referred to it as frequently as in the positive condition; that is, participants did not simply ignore the negative social information.

Another possibility is that there might be two types of participants: one using negative social information efficiently, and the other performing even worse than asocial learners due to maladaptive imitation of unsuccessful exemplars; these two effects might have canceled each other out. However, this is again unlikely. The standard deviation of the scores in the negative condition was not particularly large compared to that in the asocial condition (see Fig. 3b). The F-test for equality of variance showed that the variance in the final trial’s performance was not significantly different between the negative and asocial conditions in all three seasons: Season 1 (*F*_39,39_ = 0.89, *p* = 0.728), Season 2 (*F*_39,39_ = 0.71, *p* = 0.292) and Season 3 (*F*_39,39_ = 0.86, *p* = 0.639). Furthermore, the distribution of the individual scores in the negative condition largely overlaps with that in the asocial condition (see Fig. S5).

## Agent-based computer simulation

We conducted agent-based simulations to predict how well artificial learning algorithms would perform in the setting of our experiment. The details of the simulations are given in the Supplementary Material (B. Details of the computer simulation). We followed the method of Mesoudi and O’Brien^40^, who also simulated their own experiment^39^ with artificial learning strategies^40^. The simulation code was coded in C++ by YN. Our simulations were identical to our experiment in the following respects: the shape of the fitness landscape in each of the three seasons, the initial values of the arrowhead attributes, the number of hunting events, the timings of the opportunities for social learning, and the distributions of error terms. Mesoudi and O’Brien ignored the two discrete attributes (color and shape) to simplify their simulations, and we adopted the same approach. Below, we explain the learning algorithms used in the simulations.

We adopt the ‘Win-Stay Strategy’ as an individual learning algorithm, following Mesoudi and O’Brien^40^. The algorithm works in a similar way to Skinnerian conditioning^40^. Each agent memorized its own score in the previous trial and the direction of modification (either + or −) for each of the three attributes. In each trial, each agent randomly chose one of the three attributes (height, width, and thickness) and modified the value of the chosen attribute by *L*_*i*_ units in the direction memorized and then went hunting. As in the previous simulation study^40^, we set *L*_*i*_ = 5, which is the median of the empirical modification sizes observed in Mesoudi and O’Brien’s experiment^39^ and in our experiment. If the score did not decrease compared to the previous trial, the agent kept the direction of modification unchanged for the focal attribute; otherwise, the agent changed it. Note that the sign of the direction of change is updated for one attribute in each trial. The agents kept following this algorithm through all three seasons unless they were given opportunities for social learning.

As in the experiment, we also considered social learning conditions, where social learning opportunities came once in every three trials in Seasons 2 and 3. Three hunters appeared, each of whom brought an arrowhead slightly modified from the focal agent’s arrowhead in the previous trial with respect to only one of the three continuous attributes. Each attribute was modified by exactly one hunter, so that agents could observe hunting results of three arrowheads modified in different dimensions. We considered several simple social learning algorithms that differ in the extent to which agents were influenced by the other hunters (for the details of the algorithms, see Supplementary Material B3.2 Social learning algorithm). Below we only report the results for two of such algorithms, one for the positive condition and the other for the negative condition; other social learning algorithms, however, yielded qualitatively similar results, as discussed in the Supplementary Material.

In the algorithm for positive observational learning, which we call *copy-successful-individuals (CSI)*, the agents copy all the modifications made by other hunters at once. Given that an agent of this type combines all three modifications, and each of the other hunters modifies only one attribute at a time, the resulting arrowhead must be superior to any of the arrowheads brought by the other hunters (unless the agent already found the optimal values). On the other hand, the algorithm for negative observational learning, which we call *Reverse*, is slightly more complicated. In this algorithm, agents change the values of the attributes in the opposite directions to the modifications made by other hunters. More specifically, they modify the value of attribute *j* by − *L*_*s,j*_, given that the corresponding hunter modifies the attribute by *L*_*s,j*_. Note that the learning rules analogous to our *CSI* were addressed in the literature ^17,28,44^. We do not know of any equivalents of *Reverse* addressed in the literature, although it is a natural variant of *CSI* in that it is equivalent to *CSI* in all respects but the signs of the modifications made to the attributes.

The simulation result of *CSI* closely resembled the experimental result of the *positive* condition (see Fig. 1), where *CSI* agents consistently outperformed asocial agents. However, the simulation result of *Reverse* was not necessarily obvious in light of the corresponding experimental result. In Season 2, *Reverse* agents outperformed asocial agents in the first 10 trials or so, but later, the difference in performance between the two types of agents vanished. A similar pattern was observed in the experiment, although negative observational learners eventually performed worse than asocial learners. The simulation result for Season 3 attested that negative observational learners could in theory outperform pure asocial learners. Furthermore, as shown in the Supplementary Material, we found that other algorithms of negative observational learning, which exploit much less social information, outperformed asocial learning (see Fig. S4). Despite these theoretical results, the experimental results did not show evidence that participants advantageously exploited social information about unsuccessful individuals.

## Discussion

In our experiment, individuals given the opportunities for positive observational learning performed markedly better than those who were given only opportunities for asocial learning. Conversely, negative observational learning did not show any appreciable effect on performance when compared to pure asocial learning. An additional analysis showed that participants in the negative condition referred to social information as much as those in the positive condition did, indicating that negative social information was not just ignored. Computer simulations showed that rather simple algorithms could exploit negative social information to increase overall performance. Thus, the experimental results contradicted theoretical predictions, suggesting that humans are not as rational or intelligent as the simulations presumed.

One possible factor that may have influenced the present result is informational/environmental uncertainty to decision makers often discussed in an adaptive decision framework^45,46^. In terms of our experiment, uncertainty comes from the lack of information on the shape of the fitness landscape and noise in performance scores displayed to participants. Under such uncertainty, copying the arrowhead of successful individuals almost ascertains an increase in the performance, regardless of how complex the fitness landscape would be, theoretically speaking, inasmuch as the random noise is relatively small^47,48^. In contrast, exploiting negative social information could help narrow the search space but does not ensure increased performance. Thus, exploiting negative information should be challenging for humans, bounded rational agents in both informational availability and cognitive capacities (memory and calculation^45^). Further, a previous empirical study suggested that, when useful social information is given, participants invested less cognitive effort in information processing to infer the rules behind the task^49^. Thus, it is possible that in our experiment negative social information interfered with asocial learning effort of participants; they might have enjoyed the benefits of negative social information, but this positive effect, if any, was not large enough to exceed the negative side effect (interference with asocial learning). Thus, given these cognitive limitations of humans, negative social learning might play only a limited role in the cultural evolution of technologies at least in complex situations where multiple dimensions of artifacts are involved and/or the performance of artifacts is not fully deterministic as in our experiment.

There are at least two reasons to expect that humans are generally more likely to learn from success than failure of others specifically in the technological domain. First, negative information may be inherently more difficult to process than positive information in many realistic situations. We suppose that such difficulty largely comes from inferring the structure of the fitness landscape underlying each technological problem, as discussed in the previous paragraph. Note that it is more difficult to infer the local structure of the landscape from negative information alone in a highly rugged landscape than in a smooth landscape. Negative social information would therefore be more useful in simple tasks than in complex tasks. Second, human psychology for social learning is probably innately biased toward copying. It is known that human children tend to copy not only relevant but also irrelevant actions of role models when they try to achieve a goal^7^. On the other hand, chimpanzees (*Pan troglodytes*) copy only relevant actions in the same situation. Arguments on the adaptive meanings of this ‘overimitation’ in humans have not settled down yet. One plausible explanation is ‘cultural Pascal’s wager’^50,51^; i.e., apparently irrelevant actions should be copied if the potential risk of overlooking important actions, which appear to be irrelevant but actually are not, is larger than the cost of copying and doing irrelevant actions. Note that the abovementioned two factors are not mutually exclusive and may even be intertwined. If positive information is generally useful compared to negative or other kinds of social information, natural selection would favor psychological bias toward copying. However, it is probably premature to argue that faithful copying alone explains the uniqueness of human cumulative culture. For example, a previous study on causal understanding showed that 3-year-old children were already able to exploit negative social information to increase performance^36^. Note, however, that the children could exploit negative information only when it was provided together with positive information. This latter result seems to be consistent with our conclusion that negative information is less likely to be exploited than positive information. Further research would be necessary to evaluate the extent to which human learning is inclined toward copying.

As argued above, our experiment requires participants to infer the local shape of the fitness landscape at least in the asocial and negative social learning conditions. Information for the inference mostly comes from trials and errors by self or others. The task does not require causal understanding in a strict sense because there are no principles underlying the relationship between artifact designs and performance. Therefore, there is still room for negative social learning to function usefully if an experimental setup allows participants to derive underlying principles based on others’ experiences and extrapolate the result to yet unexplored combinations of attribute values. The role of causal understanding in human cultural evolution is under debate. One experimental study suggested that causal understanding may not be a prerequisite for cumulative improvements of technology^52^; however, another study using the same experimental framework showed that understanding of technological structure fostered the cumulative improvement^53^ of performance. Further research on cognitive processes^15,54,55^ involved in cultural transmission would be necessary.

It has been argued that learning from negative examples is just as important as learning from positive examples in the field of social sciences, such as education^56^ or management science^57^, as well as in machine learning^58^. Although an experimental study in management science showed that participants could learn from others’ mistakes, it also insisted that learning from others’ failures had been neglected in the discipline^57^. In fact, an organization can learn a lot from its members’ failures, but a failure is not often systematically exploited (e.g., ending in a ‘witch-hunt’); one exception may be the military, which employs systematic practice for learning from others’ failures^59^. Together, these examples may indicate that copying or learning from positive social information is entrenched in human cognition and is easy to exercise, while its negative counterpart, learning from negative examples, requires systematic practices despite its rationality.

## Methods

### Ethics

The research was conducted in accordance with the protocol approved by the ethics committee of Kochi University of Technology (ID: 202) and in accordance with the ethical guidelines of Kochi University of Technology.

### Participants

A total of 120 students (undergraduate and graduate, aged 18 to 26 years; gender: 62 females, 57 males, 1 no response) in Kochi Prefecture participated. One participant who did not complete the postquestionnaire was excluded from the postquestionnaire analysis but was included in the main analysis. All participants were recruited via the online recruitment system. Each of them received 700 JPY as the basic participation fee plus an additional reward up to 1000 JPY according to the score that the participant earned through virtual hunting. Informed consent was obtained from all participants.

### Materials

All the participants conducted the experimental task with computers surrounded by three-way partitions. The experimental program of the virtual arrowhead task was reconstructed with Visual Basic (Visual Studio 2019), referring to the C++ source code provided by the author of the original program^39^ and the corresponding executable file downloaded from his website (http://alexmesoudi.com/resources/, retrieved September 2019). We kept the overall design of the experimental program identical to the original studies ^39,41^, except for two points: (1) in the original studies, the instructions were embedded in the program, while we presented the instructions separately; so that the participants could refer to the instructions throughout the experiment whenever they wanted; (2) more importantly, we modified the way social information was displayed. We explain this modification in detail in the “Design” section.

### Procedure

Upon arrival, participants filled out the consent and medical check forms (to exclude those who were potentially infected by COVID-19; none of participants were excluded in this process). After completing the forms, the participants received the instructions for the experiment. One of the experimenters read out the instructions with the aid of slides projected onto a screen in front of the participants. The instruction slides were also printed and distributed to the participants as handouts, which they could refer to throughout the experiment. Then, to ensure that participants understood the instructions correctly, every participant filled out a printed prequestionnaire, and then the answers were immediately checked by the experimenters (Table S2). When participants failed to answer some of the questions correctly, they received as much additional explanation as necessary on an individual basis so that every participant was eventually able to answer all the questions correctly. Once all participants finished the prequestionnaire, they started the main experimental task on the computers. Upon completing the main task, each participant filled out a postquestionnaire and subsequently received their payment. The whole experiment lasted an average of 40 min per session, but no more than 60 min.

### Experimental task

**Figure 4:**
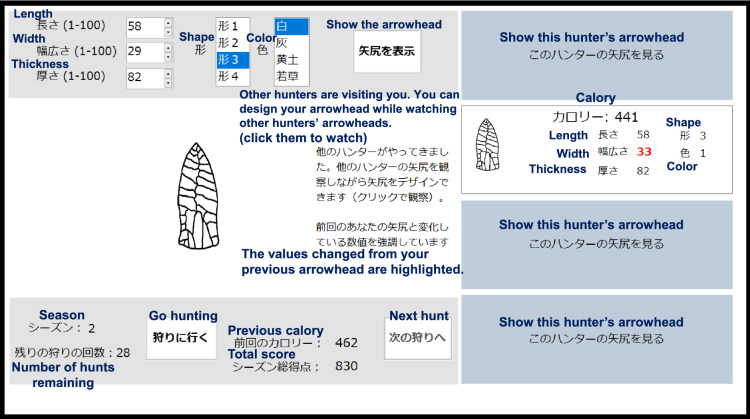
A sample screenshot of the experimental screen (negative social learning condition). English translations of labels are added.

In the main task, participants repeatedly designed a virtual arrowhead and earned scores in a learning-while-doing manner (Fig. 4). In each trial, participants set the values for five attributes of an arrowhead. For three of the five attributes (length, width, and thickness), the values might take integer values between 1 and 100, while the other two attributes (shape and color) were discrete traits with four categorical options each. Each time participants changed the values of the attributes, they could check the appearance of the resulting arrowhead by clicking the ‘Show the arrowhead’ button. Once participants were satisfied with the arrowhead design, they clicked the ‘Go hunting’ button to submit the design and obtained its score displayed in units of hypothetical ‘calories’. The score, which represented the performance of the arrowhead designed by the participant, ranged from 1 to 1000 for every single hunting opportunity. We used the same formula as in the unimodal setting of original studies^17,40,41^ (Formulas (S1), (S2)) to compute the score for each arrowhead design. Specifically, this formula assumes a predefined fitness landscape (i.e., a mapping from the designs to the scores), in which the attributes contribute additively and independently to the expected resulting score. However, the actual score earned by each participant was perturbed by a small noise, which was normally distributed ($\sim$ *N* (0, 5^2^)), so that the shape of the fitness landscape was not obvious from the observed scores alone. Participants were informed in the instructions about the presence of the noise, although not about the shapes of the noise function and the fitness landscape. The payment was calculated based on the performance with this noise. Participants were encouraged in the instructions to obtain as many calories as possible and were also monetarily incentivized by being informed that they would gain additional JPY equivalent to the average score earned per hunting event.

Each session of the experiment consisted of three seasons (Seasons 1–3), with 30 trials each. The fitness landscapes differed between seasons but stayed unchanged within each season, about which participants were also informed (understanding of this was confirmed in the prequestionnaire).

### Postquestionnaire

In the postquestionnaire, each participant subjectively rated the importance of each of the five attributes for the performance of the arrowhead on a 7-point scale (Table S3). In addition, in conditions where social information was provided, an additional question was asked about the subjective evaluation of the usefulness of the social information.

### Design

A between-subjects design was adopted, where each participant was randomly assigned to one of three conditions: *positive*, *negative*, and *asocial* conditions. In the positive and negative conditions, participants had opportunities for positive and negative observational learning, respectively, whereas in the asocial condition, they had no such opportunities. In Season 1, participants conducted the virtual arrowhead task without any social information, irrespective of the experimental conditions. This season helped participants get used to the task and allowed us to make fair comparisons of participants’ basic skills across different conditions. In Seasons 2 and 3, participants in the positive and negative conditions had opportunities to observe the arrowheads designed by four hypothetical ‘other hunters’, which were artificially generated. The opportunities for observation were given once in every three trials when they designed the arrowhead. In the positive (negative) condition, the scores of the other hunters were always higher (lower) than that of the participant in the previous trial. In the asocial condition, participants continued to work on the same task without social information as in Season 1, except that the fitness landscapes differed between seasons.

The arrowheads of the other hunters displayed to each participant were generated based on the arrowhead designed by the focal participant in the previous trial (let us call this the ‘reference’ arrowhead). Specifically, each of the four other hunters slightly modified one of the three quantitative attributes, i.e., length, width, and thickness. In the positive condition, the absolute size of the modification was normally distributed, with mean = 10 and *SD* = 10, and the sign of the modification was always chosen to increase fitness compared to the reference arrowhead. Then, like participants, a normally distributed noise ($\sim$ *N* (0, 5^2^)) was added to the fitness before it was shown to the participant. This addition of noise occasionally made the fitness of the other hunter’s arrowhead lower than the reference. In this case, we resampled both the attribute value and the perception error. Resampling was repeated a maximum of 10,000 times until we found a combination of an attribute value and noise that made the other hunter’s arrowhead better than the reference in terms of both true and perceived performance. In case this procedure could not find such a combination, we set the other hunter’s arrowhead to the same as the reference (this typically occurred when a participant was at the maximum of the landscape). Participants were told that other hunters would imitate the participant’s arrowhead in the previous trial and bring only improved ones. Every participant understood that the arrowheads of other hunters were generated based on, and always superior to, the participant’s own arrowhead in the previous trial, as we confirmed in the prequestionnaire. The negative condition underwent a similar procedure, but unlike in the positive condition, the sign of each modification was chosen to *reduce* the performance of the arrowhead. Likewise, resampling for each hunter was repeated until we obtained an arrowhead which was inferior to the reference in terms of both true and perceived fitness or the maximum repeat count (10,000) was reached; in the latter case, the other hunter’s arrowhead was set to the reference as in the positive condition. Participants were told that other hunters would imitate the participant’s arrowhead in the previous trial and bring only ones with reduced performance. Again, every participant understood how other hunters’ arrowheads would behave, as we confirmed in the prequestionnaire.

In the positive and negative conditions, the information regarding the arrowheads of other hunters (appearance, score, and the values of the five attributes) was concealed behind four gray panels arranged vertically on the right side of the display. The information of each hunter’s arrowhead was revealed only when the left button of the mouse was pressed on the corresponding panel and held down; it was hidden again when the button was released. Thus, a participant could see only one of the four arrowheads at a time, but as long and as many times as the participant wanted. When the information on the arrowhead was shown, the value changed was highlighted with colored characters in bold font to reduce the participants’ cognitive burden. In the asocial condition, no social information was displayed.

## Supplementary Information

Supplementary Information 1.

Supplementary Information 2.

Supplementary Information 3.

## Data Availability

The main experimental dataset and reproductive analysis code are attached as Supplementary Files. All materials (including instructions, computer simulation code, experimental program, datasets, and analyses codes) are available at https://github.com/YNakawake/projectile_neg.
