# Supplementary material for: Negative observational learning might play a limited role in the cultural evolution of technology
Source: Sci Rep. 2022 Jan 19;12:970. doi: 10.1038/s41598-022-05031-2 (PMC8770688; doi:10.1038/s41598-022-05031-2)
Supplement: Supplementary file 1 — Supplementary Information 1. [file 41598_2022_5031_MOESM1_ESM.docx]

Electronic Supplementary Materials:

‘Negative observational learning might play a limited role in the cultural evolution of technology’

by Yo Nakawake and Yutaka Kobayashi

The file includes:

A. Shape of the fitness landscape

- Formula S1
- Formula S2
- Table S1

B. Details of the computer simulation

B1. Basic framework

B2. Individual learning algorithm

- Figure S1

B3. Social learning: condition and algorithms

B3.1 Condition

B3.2 Social learning algorithms

- Figure S2
- Figure S3

B4. Results

- Figure S4

C. Item list of prequestionnaire

- Table S2

D. Item list of postquestionnaire

- Table S3

E. Supplementary Figures

- Figure S5
- Figure S6

**A. Shape of the fitness landscape**

The formula to calculate the fitness landscape was identical to the unimodal version of Mesoudi & O’Brien (2008b) ^1^. That is, the performance (fitness) of the arrowhead *W* (0 ≤ *W* ≤ 1000) was given by a weighted sum of the contributions of four attributes (length, width, thickness, and shape), as follows:

*W* = 1000 (0.275 *W_L_* + 0.25*W_W_* + 0.35 *W_T_* + 0.125 *W_S_*), (S1)

where *W_L_*, *W_W_*, *W_T_*, and *W_S_* represent the fitness contributions of length, width, thickness, and shape, respectively. The contribution of color is not included in the formula, given that color has no influence on performance.

For continuous attributes (length, width, and thickness), each fitness contribution (resp. *W_L_*, *W_W_*, and *W_T_*) is a Gaussian function of the difference between the optimum of the fitness landscape (resp. *O_L_*, *O_W_*, and *O_T_*) and the value participants input (resp. *X_L_*, *X_W_*, and *X_T_*). For example, in the case of length, *W_L_* is given by

*W_L_* = exp [−(*X_L_*/100 − *O_L_*/100)^2^ /2*σ*], 　(S2)

where the length *X_L_* of the arrowhead and its optimal value *O_L_* are both scaled by the common upper limit 100 for normalization. The optimal values of attributes for each season are shown in Table S1. We set the dispersion parameter *σ* to 0.025 for all three continuous attributes. For discrete attributes (i.e., shape), *W_S_* took one of four values (1, 0.9, 0.66, 0.33), depending on the specific shape of the arrowhead (Shapes 1, 2, 3, and 4). The different values were assigned to each type of shape depending on season (see Table S1). Finally, the participant received the performance information only after a random noise ~*N* (0, 5^2^) was added to the true performance *W*; the participant was not informed of the value of *W* itself. This perceived performance with random noise was used to calculate the payment to participants (or was used as feedback to agents in the computer simulation).

Table S1. Parameter sets used in the experiment

|  | Season 1 | | Season 2 | | Season 3 | |
| --- | --- | --- | --- | --- | --- | --- |
|  | Initial | Optimal | Initial | Optimal | Initial | Optimal |
| Length | 52 | 30 | 58 | 36 | 61 | 39 |
| Width | 22 | 63 | 73 | 4 | 79 | 17 |
| Thickness | 75 | 34 | 82 | 57 | 38 | 84 |
| Shape | 2 | [3, 4, 2, 1] | 3 | [1, 2, 3, 4] | 2 | [1, 3, 2, 4] |
| Color | 3 |  | 1 |  | 1 |  |

*Note.* Numbers in brackets [] are the indices of the four shapes sorted in descending order of fitness contribution (*W_S_* = 1, 0.9, 0.66, 0.33, resp.).

**B. Details of the computer simulation**

**B1. Basic framework**

The basic framework of our simulation model was identical to Mesoudi & O’Brien’s (2008b) model ^1^, which intended to simulate the results of their own experimental study (Mesoudi & O’Brien 2008a) ^2^. In this framework, agents explored a cultural fitness landscape of virtual arrowheads in learning-while-doing manners. The fitness of the arrowheads was given by a weighted sum of the contributions from three attributes (length, width, and height; for details, see Table S1 and Formula S1), where each of the attributes takes an integer value between 1-100. In 30 hunting trials, agents had the opportunity to modify their arrowheads and obtain fitness values as feedback, which ranged from 1 to 1000 (i.e., calories obtained by hunting). However, as in the experiment, the success of hunts was influenced by random noise unrelated to the design of the arrowheads. The agents could only obtain the fitness information disturbed by random noise ~*N* (0, 5^2^) and not the true fitness value itself.

At the beginning of the simulation (i.e., the first trial), the agent received a predesignated arrowhead as the starting point. As in our experiment, the attribute values of the predesignated arrowhead (i.e., initial attribute values) were adopted from one of the parameter settings provided in the previous experimental study (Mesoudi & O’Brien, 2008a) ^2^. For the first trial, agents had to hunt with the predesignated arrowhead; thus, the fitness in the first trial was the same regardless of conditions and learning strategies. From the second hunt, agents modified their arrowheads based on the following individual learning or social learning algorithm.

**B2. Individual learning algorithm**

The individual learning algorithm was identical to that in Mesoudi & O'Brien (2008b) ^1^ (see Figure S1). In each modification phase, agents randomly chose one of three continuous attributes (length, width, and thickness) and modified its value by 5 units. For example, if the width was chosen and its current value was 50, the value was modified to either 45 or 55. The sign of modification was determined based on the agent’s memory. Each agent memorized an array of three entries, each of which stored the sign (‘+’ or ‘−’) of modification for the corresponding attribute (length, width, and thickness). For the first trial, the signs were randomly determined. An agent updated its memory through introspection of the latest modification and its fitness consequence. For example, suppose that an agent ‘decides’ to modify the width, and the sign currently memorized for width is ‘−’. If the current width is 50, then the width is reduced to 45; in other words, the width is changed by 5 units in the direction of ‘−’. If the fitness increased compared to the previous hunt as a consequence of this change, then the agent kept the sign of the modification for width unchanged. However, if the fitness decreased, then the agent flipped the sign for width in the memory (i.e., switched it to the opposite direction). In this way, an agent continued to explore the trait space in the same direction while the outcome was successful but reversed the direction as soon as the outcome was unsuccessful.

Note that the size of the modification (i.e., 5) and the number of attributes were identical to those in Mesoudi & O’Brien (2008a) ^2^. The rationale behind these settings was based on the behavioral experiment (Mesoudi & O’Brien, 2008a) ^2^. According to Mesoudi & O’Brien (2008a, b) ^1,2^, the participants tended to change the value of each attribute by 5 units in the behavioral experiment. Furthermore, Mesoudi & O’Brien (2008b) ^1^ reported that these values were optimal in their simulation study.


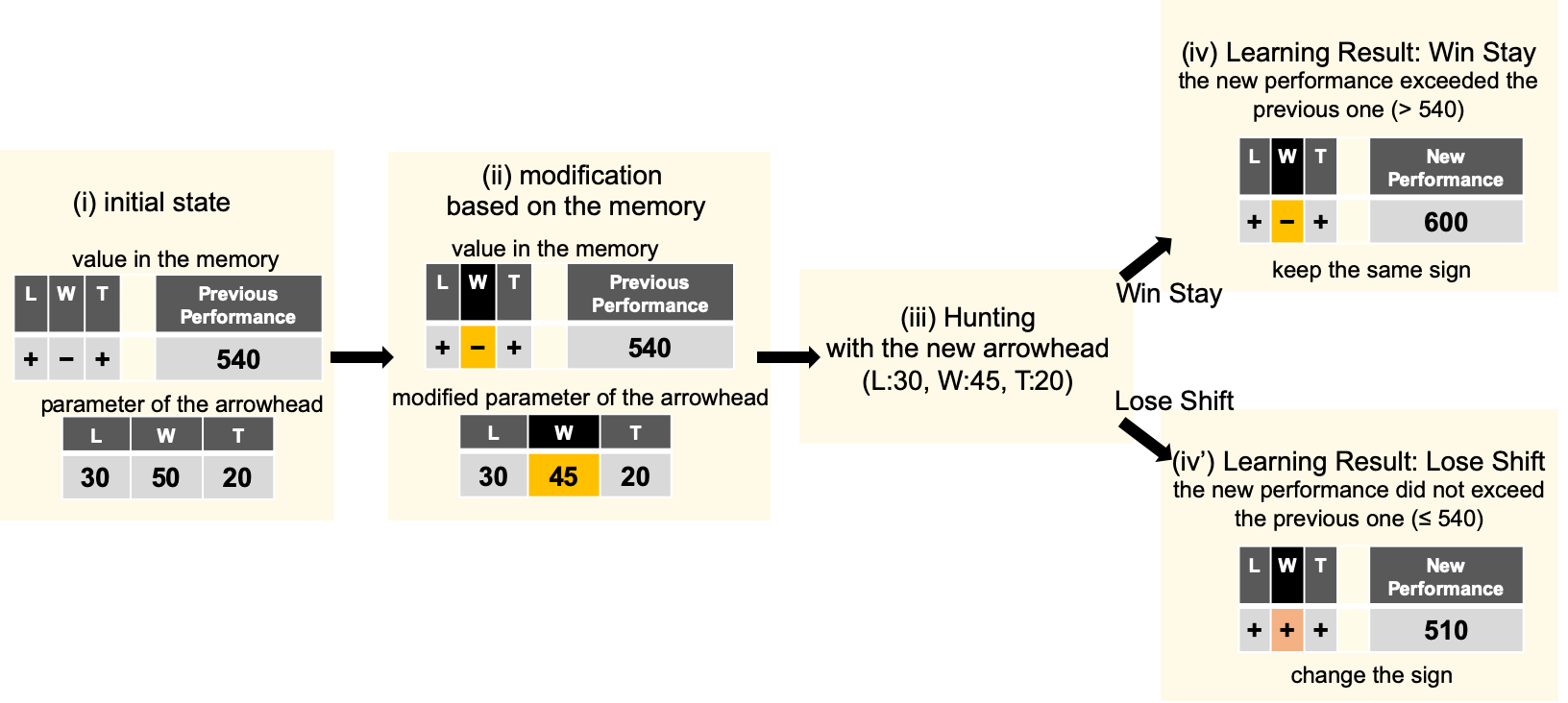


Figure S1. Schematic illustration of the individual learning algorithm in our simulation model. ‘L’, ‘W’, and ‘T’ represent length, width, and thickness, respectively.

As Mesoudi & O’Brien (2008b) ^1^ noted, this learning algorithm works in a similar manner to the ‘win-stay, lose-shift’ rule ^3^ and Skinnerian conditioning. Although this algorithm is rather simple, the results thoroughly explained the behaviors of participants observed in their own experiment, whose setups were almost identical to those of the model (Mesoudi & O’Brien, 2008a) ^2^. Furthermore, in studies of adaptive decision making ^4^, it is often the case that simple learning algorithms fit well with behavioral data compared to other rules requiring more cognitive resources ^5^.

**B3 Social learning: conditions and algorithms**

**B3.1 Conditions**

As in the experiment, we consider three conditions: the positive, negative, and asocial learning conditions. In the positive (negative) condition, agents could socially learn only from exemplars with higher (lower) fitness. In the asocial condition, agents had no opportunity to observe exemplars. Agents had social learning opportunities once in every three trials (i.e., in trials 3, 6, 9, 12, …, 30). Each exemplar’s arrowhead was generated by modifying one of the three attributes of the arrowhead designed by the focal agent in the previous trial (i.e., reference arrowhead), where different exemplars modified different attributes. The size of modification *s* was randomly sampled from the normal distribution *N* (*μ*, *σ*^2^). The mean *μ* was set as either 10 or −10 with equal probabilities, and the standard deviation *σ* was set as 10. In the positive (negative) condition, resampling was repeated at most 10,000 times until *s* was found to make the exemplar’s arrowhead superior (inferior) to the reference arrowhead in terms of both true and perceived fitness, where the latter includes the effect of perception error. When the count of resampling reached its maximum 10,000, the hunter’s arrowhead was set to the reference arrowhead.

**B3.2 Social learning algorithms**

We considered three types of social learning algorithms or strategies. In the first algorithm, *Memory Only*, social learning only influences the agent’s memory for modification signs and does not immediately influence the arrowhead. Conversely, the latter two algorithms do not alter memory but instead affect the arrowhead immediately. Note that opportunities for individual learning are equally given to all types of agents, regardless of whether social learning opportunities are given or not. That is, individual learning opportunities are given to agents even in rounds when social information is provided. This is a rational assumption, given that in our experiment, the presence of social information, at least in theory, never reduces the opportunities of individual learning; however, the two kinds of information might interfere with each other at a cognitive level (see the assumption of *Memory Only* below).

***Memory Only.*** This algorithm only updates memory for modification signs but does not immediately change the arrowhead. In the positive condition, agents equipped with this algorithm change the states of the three memory slots in accordance with the signs of the differences between the exemplars’ values and their own. For example, if an exemplar’s arrowhead is longer (wider, thicker) than their own, the agents set the memory slot for length (resp. width, thickness) to ‘+’. In the negative condition, the opposite is the case, so that they set the signs in memory to the opposites of the signs of the differences between exemplars’ values and their own. Note that any two of the three exemplars do not modify the same attribute simultaneously; hence, signs obtained from the three exemplars never contradict each other. Thus, most times, agents’ memories were updated to match the social information. However, if the social information contradicts the previous result of individual learning of previous individual learning (e.g., the social information is ‘+’, while the individual information is ‘−’), agents randomly pick either ‘+’ or ‘−’ (coin flip model ^6^). In this learning algorithm, agents are given an extra opportunity to update their memory but not to touch the arrowhead. Note that here, agents kept modifying the arrowhead at the same pace as pure asocial learners, i.e., 5 units in one attribute per trial.

***Variable Stride.*** This is the algorithm reported in the main text and consists of two subtypes: *Copy-Successful-Individuals (CSI)* for the positive condition and *Reverse* for the negative condition. The results of these algorithms are described in detail in the main text. In CSI, the agents copy all the modifications made by exemplars at once (see Figure S2). On the other hand, agents of *Reverse* modify the value of attribute *j* by *-L_s,j_*, given that the corresponding exemplar modified the attribute by *L_s,j_* (see Figure S2). Note that agents are unlimited to change units for dimension in this algorithm, and social information gave a higher impact on the agent’s arrowhead.

Figure S2. Schematic diagram showing how *Variable Stride* works.

***Fixed Stride***. This algorithm again includes two subtypes: fixed-stride versions of CSI and *Reverse*. Agents endowed with the fixed-stride CSI modify their arrowheads in the same directions as those of *CSI* but only by 5 units (see Figure S3). Likewise, the fixed-stride version of *Reverse* modifies the arrowhead attributes in the same directions as *Reverse* does but only by 5 units. In most cases, both of these short-stride types modified all three attributes by 5 units each, which amounted to 15 units in total (unless the boundaries of attribute values were reached). Here, the impact of social learning is generally weaker than in *Variable Stride* and stronger than in *Memory Only*.

Figure S3. Schematic diagram showing how *Fixed Stride* works.

***Other possibilities***. Above, we proposed three types of algorithms in which social learning could have different levels of impact: low (*Memory Only*), middle (*Fixed Stride*), and high (*Variable Stride*). Given that *Memory Only* affects memory and *Fixed/Variable Stride* affects the physical attributes of an artifact, we could combine them to make new algorithms that affect both memory and physical attributes*.* Alternatively, we could add several other potential algorithms. However, given that our major objective of the simulation was not to explore the best or intelligent algorithms but rather to *interpret* the results of the experiment, we confined ourselves to those rather simple algorithms. Of course, it is worth improving the algorithms to reproduce experimental results more faithfully, but we consider it out of the scope of the present work.

**B2.1. Results**

The simulation results showed that agents performed nearly equally well in both positive and negative conditions when *Memory Only* or *Fixed Stride* was adopted for social learning algorithms (Figure S4). Only when *Variable Stride* was adopted did agents in the positive condition outperform those in the negative condition. The results of the negative condition with the *Memory Only* algorithm showed that even minimal use of negative social information could still benefit overall performance.


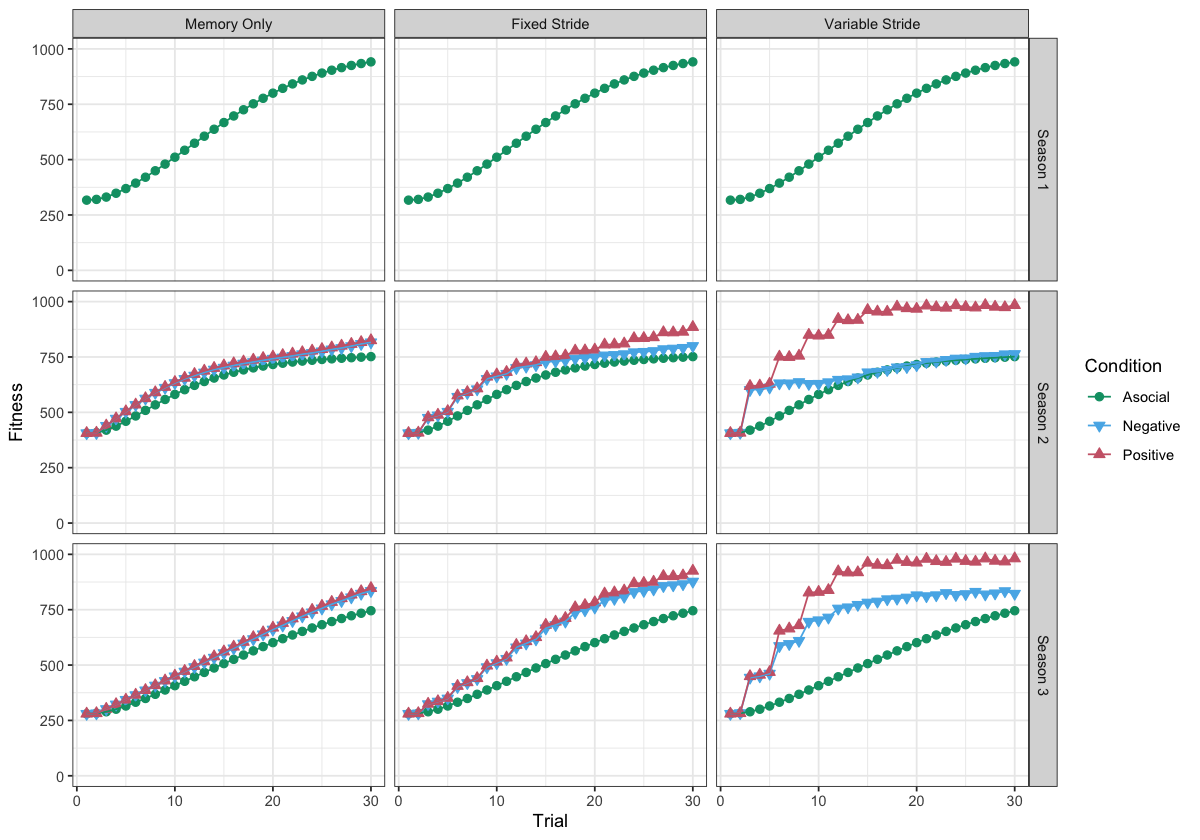


Figure S4. The results of computer simulations with different social learning algorithms (left column: *Memory Only*, center column: *Fixed Stride*, right column: *Variable Stride*). The rows correspond to seasons. The result for *Variable Stride* (right column) is identical to Figure 2 (b) in the main text.

**C. Item list of the prequestionnaire**

Table S2. Item list of the prequestionnaire (translated into English).

| No. | Question | Answer |
| --- | --- | --- |
| 1 | Across three seasons, the superior shapes of arrowheads are constant. | False |
| 2 | Other hunters (those coming to show their arrowheads to you) made arrowheads referring to your arrowhead. | True |
| 3 | It is possible that other hunters (those coming to show their arrowheads to you) come to show arrowheads that yield more calories than your previous arrowhead. | Negative: False  Positive: True |
| 4 | It is possible that other hunters (those coming to show their arrowheads to you) come to show arrowheads that yield fewer calories than your previous arrowhead. | Negative: True  Positive: False |
| 5 | Within the same season, environments can change, and the beneficial shape of arrowheads for hunting can change. | False |
| 6 | Even though you keep hunting with an arrowhead of the same shape, the calories can vary with a random error. | True |
| 7 | There is no difference in ability among hunters (calories can vary due to a random error but not due to hunting ability). | True |

Note. All items are binary choice questions (True/False). Items No. 2, 3, 4, 5, and 7 were not shown to participants in the asocial condition. For No. 2 and 3, answers differ depending on the experimental condition.

**D. Item list of the postquestionnaire**

Table S3. Item list of the postquestionnaire (translated into English) and mean rating scores (7-point Likert scales, 1: Not very import, 4: Neutral, 7: Very important).

| Items | Mean rating score (*SD*) | | |
| --- | --- | --- | --- |
|  | asocial | positive | negative |
| How important is ‘Length’ for gaining high calories? | 5.5 (1.12) | 5.9 (0.98) | 5.2 (1.40) |
| How important is ‘Width’ for gaining high calories? | 5.5 (1.45) | 5.8 (1.31) | 6.1 (1.15) |
| How important is ‘Thickness’ for gaining high calories? | 5.8 (1.06) | 6.1 (1.05) | 6.2 (0.75) |
| How important is ‘Color’ for gaining high calories? | 3.3 (1.76) | 3.1 (1.57) | 2.9 (1.54) |
| How important is ‘Shape’ for gaining high calories? | 4.9 (1.45) | 5.1 (1.36) | 4.4 (1.58) |
| How useful are the other hunters’ arrowheads?  (1:Not very useful, 7:Very useful) |  | 4.7 (1.75) | 6.5 (1.22) |

*Note.* One participant in the asocial condition who did not complete the questionnaire was excluded.

**E. Supplementary Figures**


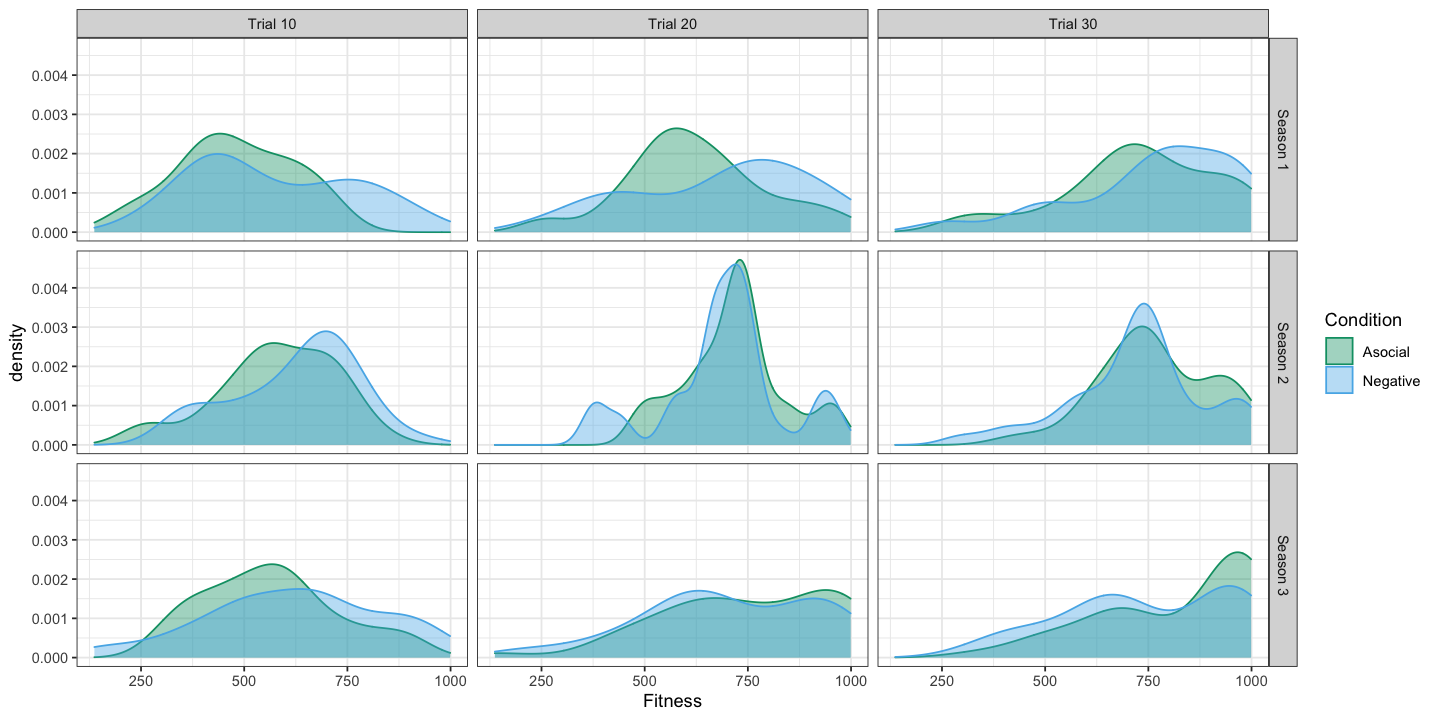


Figure S5. Kernel density plot of performance in trials 10, 20 and 30 of Seasons 1, 2 and 3. In the final trial (30) of Season 2 and Season 3, the distributions of the negative condition almost overlapped with those of the asocial conditions.

Figure S6. (a) Total looking time and (b) Total frequency of mouse clicks.

**References**

1. Mesoudi, A. & O’Brien, M. The cultural transmission of Great Basin projectile-point technology II: An agent-based computer simulation. *Am. Antiq.* **73**, 627–644 (2008).

2. Mesoudi, A. & O’Brien, M. J. The cultural transmission of Great Basin projectile-point technology I: An experimental simulation. *Am. Antiq.* **73**, 627–644 (2008).

3. Nowak, M. & Sigmund, K. A strategy of win-stay, lose-shift that outperforms tit-for-tat in the Prisoner’s Dilemma game. *Nature* **364**, 56–58 (1993).

4. Gigerenzer, G., Todd, P. M. & Group, the A. research. *Simple heuristics that makes us smart*.

5. Todd, P. M., Dieckmann, A. & Todd, P. M. Simple rules for ordering cues in one-reason decision making. in *Ecological rationality: Intelligence in the world* 1393–1400 (2012).

6. Bahrami, B. *et al.* Optimally interacting minds. *Science* **329**, 1081–5 (2010).
