# Supplementary material for: Negative observational learning might play a limited role in the cultural evolution of technology
Source: Sci Rep. 2022 Jan 19;12:970. doi: 10.1038/s41598-022-05031-2 (PMC8770688; doi:10.1038/s41598-022-05031-2)
Supplement: Supplementary file 3 — Supplementary Information 3. [file 41598_2022_5031_MOESM3_ESM.html]

main\_analysis\_code


# Table of Contents

- 0  Data Reading, Preparation
  - 0.1  Pakages
  - 0.2  Color cordination
  - 0.3  Reading Data
  - 0.4  Aggregating 'Looking Time' and 'Frequency of Mouse-clicks'
  - 0.5  Making 30 trialsdevide into 3 blocks
- 1  Visualization
  - 1.1  Fitness
    - 1.1.1  Mean (By Condition)
    - 1.1.2  SD
    - 1.1.3  Median
      - 1.1.3.1  Median + Q1 & Q3
  - 1.2  Distribution of fitness
    - 1.2.1  negative v. asocial
    - 1.2.2  positive v. asocial
  - 1.3  Social Infomation
    - 1.3.1  Looking Time
    - 1.3.2  Click Frequency
  - 1.4  Parameters Shift
    - 1.4.1  Length
    - 1.4.2  Width
    - 1.4.3  Thickness
- 2  How participants have changed parameta?
  - 2.1  Preparation
  - 2.2  Number of changes in dimention for each trial
    - 2.2.1  Mode and Median
- 3  t-test
  - 3.1  To calculate Block Mean
    - 3.1.1  Calculate Overall Performance
    - 3.1.2  Making into tidy data
- 4  F-test (equal variance)
  - 4.1  season 1
  - 4.2  season 2
  - 4.3  season 3

## Data Reading, Preparation¶

### Pakages¶

In [1]:

```
library(stringr)
library(dplyr)
library(reshape)
library(ggplot2)
library(readr)
#library(lsa)
cosine_sim <- function(a, b) crossprod(a,b)/sqrt(crossprod(a)*crossprod(b))

# Colour-Item
library(viridis)
library(scales)
library(rcartocolor)

#effect siez
library(effsize)

library(psych)
```

```
Attaching package: ‘dplyr’


The following objects are masked from ‘package:stats’:

    filter, lag


The following objects are masked from ‘package:base’:

    intersect, setdiff, setequal, union


Attaching package: ‘reshape’


The following object is masked from ‘package:dplyr’:

    rename


Loading required package: viridisLite


Attaching package: ‘scales’


The following object is masked from ‘package:viridis’:

    viridis_pal


The following object is masked from ‘package:readr’:

    col_factor


Attaching package: ‘psych’


The following object is masked from ‘package:effsize’:

    cohen.d


The following objects are masked from ‘package:scales’:

    alpha, rescale


The following objects are masked from ‘package:ggplot2’:

    %+%, alpha
```

In [2]:

```
sessionInfo()
```

```
R version 4.0.3 (2020-10-10)
Platform: x86_64-apple-darwin17.0 (64-bit)
Running under: macOS Catalina 10.15.7

Matrix products: default
BLAS:   /Library/Frameworks/R.framework/Versions/4.0/Resources/lib/libRblas.dylib
LAPACK: /Library/Frameworks/R.framework/Versions/4.0/Resources/lib/libRlapack.dylib

locale:
[1] ja_JP.UTF-8/ja_JP.UTF-8/ja_JP.UTF-8/C/ja_JP.UTF-8/ja_JP.UTF-8

attached base packages:
[1] stats     graphics  grDevices utils     datasets  methods   base     

other attached packages:
 [1] psych_2.0.12      effsize_0.8.1     rcartocolor_2.0.0 scales_1.1.1     
 [5] viridis_0.5.1     viridisLite_0.3.0 readr_1.4.0       ggplot2_3.3.3    
 [9] reshape_0.8.8     dplyr_1.0.7       stringr_1.4.0    

loaded via a namespace (and not attached):
 [1] Rcpp_1.0.5          pillar_1.6.1        compiler_4.0.3     
 [4] plyr_1.8.6          base64enc_0.1-3     tools_4.0.3        
 [7] digest_0.6.27       uuid_0.1-4          nlme_3.1-149       
[10] lattice_0.20-41     jsonlite_1.7.2      evaluate_0.14      
[13] lifecycle_1.0.0     tibble_3.1.2        gtable_0.3.0       
[16] pkgconfig_2.0.3     rlang_0.4.10        IRdisplay_0.7.0    
[19] DBI_1.1.1           parallel_4.0.3      IRkernel_1.1.1.9000
[22] gridExtra_2.3       withr_2.3.0         repr_1.1.0         
[25] hms_1.1.0           generics_0.1.0      vctrs_0.3.8        
[28] grid_4.0.3          tidyselect_1.1.0    glue_1.4.2         
[31] R6_2.5.0            fansi_0.4.1         pbdZMQ_0.3-4       
[34] purrr_0.3.4         magrittr_2.0.1      ellipsis_0.3.2     
[37] htmltools_0.5.0     mnormt_2.0.2        assertthat_0.2.1   
[40] colorspace_2.0-0    utf8_1.1.4          stringi_1.5.3      
[43] munsell_0.5.0       tmvnsim_1.0-2       crayon_1.4.1
```

### Color cordination¶

In [3]:

```
options(repr.plot.width=6, repr.plot.height=6)
palet8  <- c("#000000", "#E69F00", "#56B4E9", "#009E73", 
                       "#F0E442", "#0072B2", "#D55E00", "#CC79A7")
display_carto_pal(5, "Safe")
CClour<-carto_pal(5, "Safe")
CPalet<-c(palet8[4],palet8[3],CClour[2])
show_col(CPalet)

CPalet
```

1. '#009E73'
2. '#56B4E9'
3. '#CC6677'

### Reading Data¶

In [4]:

```
ndf<-read.csv ("exp_data.csv")
```

### Aggregating 'Looking Time' and 'Frequency of Mouse-clicks'¶

In [5]:

```
AggLT<-as.numeric(ndf$LT1)+ as.numeric(ndf$LT2)+ as.numeric(ndf$LT3)+ as.numeric(ndf$LT4)
AggClick<-as.numeric(ndf$Click1)+ as.numeric(ndf$Click2)+ as.numeric(ndf$Click3)+ as.numeric(ndf$Click4)

ndf<-dplyr::mutate(ndf, AggLT, AggClick)


#for (i in 1:(dim(ndf)[2])){
 #   ndf[,i]<-as.numeric(ndf[,i])
#}
```

In [6]:

```
head(ndf)
```

A data.frame: 6 × 22

|  | ID | ID2 | Season | SocInfo | Trial | Length | Width | Thick | Shape | Color | ⋯ | LT1 | LT2 | LT3 | LT4 | Click1 | Click2 | Click3 | Click4 | AggLT | AggClick |
| --- | --- | --- | --- | --- | --- | --- | --- | --- | --- | --- | --- | --- | --- | --- | --- | --- | --- | --- | --- | --- | --- |
|  | <int> | <chr> | <int> | <int> | <int> | <int> | <int> | <int> | <int> | <int> | ⋯ | <dbl> | <dbl> | <dbl> | <dbl> | <int> | <int> | <int> | <int> | <dbl> | <dbl> |
| 1 | 1 | B6 | 1 | 0 | 1 | 52 | 22 | 75 | 2 | 3 | ⋯ | 0 | 0 | 0 | 0 | 0 | 0 | 0 | 0 | 0 | 0 |
| 2 | 1 | B6 | 1 | 0 | 2 | 100 | 100 | 100 | 2 | 3 | ⋯ | 0 | 0 | 0 | 0 | 0 | 0 | 0 | 0 | 0 | 0 |
| 3 | 1 | B6 | 1 | 0 | 3 | 100 | 50 | 70 | 2 | 3 | ⋯ | 0 | 0 | 0 | 0 | 0 | 0 | 0 | 0 | 0 | 0 |
| 4 | 1 | B6 | 1 | 0 | 4 | 95 | 35 | 50 | 2 | 3 | ⋯ | 0 | 0 | 0 | 0 | 0 | 0 | 0 | 0 | 0 | 0 |
| 5 | 1 | B6 | 1 | 0 | 5 | 75 | 25 | 25 | 2 | 3 | ⋯ | 0 | 0 | 0 | 0 | 0 | 0 | 0 | 0 | 0 | 0 |
| 6 | 1 | B6 | 1 | 0 | 6 | 65 | 15 | 15 | 1 | 2 | ⋯ | 0 | 0 | 0 | 0 | 0 | 0 | 0 | 0 | 0 | 0 |

### Making 30 trialsdevide into 3 blocks¶

In [7]:

```
Block<-(ndf$Trial > 0)*1 + (ndf$Trial > 10)*1  + (ndf$Trial > 20)*1
ndf<-mutate(ndf, Block)
```

## Visualization¶

### Fitness¶

In [8]:

```
labeli <- as_labeller(c("1" = "Season 1",
                         "2" = "Season 2",
                         "3" = "Season 3"))
```

#### Mean (By Condition)¶

In [9]:

```
options(repr.plot.width=5, repr.plot.height=7)
"Mean + SE"
ggplot(ndf, aes(x=Trial,y=Fitness, group = as.factor(SocInfo),  
                color = as.factor(SocInfo), fill = as.factor(SocInfo),shape = as.factor(SocInfo))) +  
ylim(0,1000)+
stat_summary(fun="mean", geom="line", alpha = 1) +
stat_summary(fun="mean", geom="point", alpha = 1, size = 2) +
stat_summary(fun = mean,
               fun.min = function(x) mean(x) - sd(x)/sqrt(length(x)), 
               fun.max = function(x) mean(x) + sd(x)/sqrt(length(x)), 
               geom = "linerange", alpha = 1)+
facet_grid(Season ~ . ,labeller = labeli) +
scale_color_manual(values = CPalet, name = "Condition",labels = c("Asocial", "Negative","Positive"))+
scale_fill_manual(values = CPalet, name = "Condition",labels = c("Asocial", "Negative","Positive"))+
scale_shape_manual(values = c(21,25,24), name = "Condition",labels = c("Asocial", "Negative","Positive"))+
theme_bw()
```

'Mean + SE'

#### SD¶

In [10]:

```
options(repr.plot.width=5, repr.plot.height=4)
ggplot(ndf[ndf$Season  != 1,], aes(x=Trial,y=Fitness, group = as.factor(SocInfo), 
                                               color = as.factor(SocInfo), fill = as.factor(SocInfo),shape = as.factor(SocInfo))) +  
stat_summary(fun= sd,  geom="line", alpha = 1) +
stat_summary(fun= sd,  geom="point", alpha = 1, size = 1.8) +
facet_grid(Season ~ . ,labeller = labeli) +
scale_color_manual(values = CPalet, name = "Condition",labels = c("Asocial", "Negative","Positive"))+
scale_fill_manual(values = CPalet, name = "Condition",labels = c("Asocial", "Negative","Positive"))+
scale_shape_manual(values = c(21,25,24), name = "Condition",labels = c("Asocial", "Negative","Positive"))+
theme_bw()+labs(y = "Standard Deviation")
```

#### Median¶

In [11]:

```
options(repr.plot.width=5, repr.plot.height=7)

ggplot(ndf, aes(x=Trial,y=Fitness, group = as.factor(SocInfo), color = as.factor(SocInfo), shape = as.factor(SocInfo), fill = as.factor(SocInfo))) +  
stat_summary(fun= "median",  geom="line", alpha = 1) +
stat_summary(fun= "median",  geom="point", alpha = 1, size = 1.8) +
facet_grid(Season ~ . ,labeller = labeli) +
scale_color_manual(values = CPalet, name = "Condition",labels = c("Asocial", "Negative","Positive"))+
scale_fill_manual(values = CPalet, name = "Condition",labels = c("Asocial", "Negative","Positive"))+
scale_shape_manual(values = c(21,25,24), name = "Condition",labels = c("Asocial", "Negative","Positive"))+
theme_bw()+
labs(y = "Median")
```

##### Median + Q1 & Q3¶

In [12]:

```
"q1, Median, q3"

q1 <-function(x){
    y<-quantile(x)[2]
    return (y)
}

q3 <-function(x){
    y<-quantile(x)[4]
    return (y)
}

options(repr.plot.width=5, repr.plot.height=7)
ggplot(ndf, aes(x=Trial,y=Fitness, group = as.factor(SocInfo), color = as.factor(SocInfo), fill =as.factor(SocInfo) )) +  
stat_summary(fun="median", geom="line", alpha = 1) +
stat_summary(fun="median", geom="point", alpha = 1) +
stat_summary(fun = median,
               fun.min = q1, 
               fun.max = q3, 
               geom = "ribbon", alpha = 0.1) +
stat_summary(fun = median,
               fun.min = q1, 
               fun.max = q3, 
               geom = "pointrange", alpha = 0.5) +
facet_grid(Season ~ .)+
scale_color_manual(values = CPalet, name = "Condition",labels = c("Asocial", "Negative","Positive"))+
scale_fill_manual(values = CPalet, name = "Condition",labels = c("Asocial", "Negative","Positive"))+
theme_bw()
```

'q1, Median, q3'

### Distribution of fitness¶

#### negative v. asocial¶

In [13]:

```
options(repr.plot.width=12, repr.plot.height=6)
Checkpoint<-(ndf$Trial == 10)|(ndf$Trial == 20)|(ndf$Trial == 30)
COND<-Checkpoint&(ndf$SocInfo != 2)

ggplot(ndf[COND,], aes(x=Fitness, group = as.factor(SocInfo), color = as.factor(SocInfo), fill = as.factor(SocInfo))) +  
geom_histogram(position = "identity", alpha = 0.4, bins = 12)+
geom_density(aes(color = as.factor(SocInfo)))+
facet_grid(Season ~ Trial)+
scale_color_manual(values = CPalet, name = "Condition",labels = c("Asocial", "Negative","Positive"))+
scale_fill_manual(values = CPalet, name = "Condition",labels = c("Asocial", "Negative","Positive"))+
theme_bw()
```

In [14]:

```
labeli_temp <- as_labeller(c("1" = "Season 1",
                         "2" = "Season 2",
                         "3" = "Season 3",
                        "10" =  "Trial 10",
                        "20" =  "Trial 20",
                        "30" =  "Trial 30"
                            ))


options(repr.plot.width=12, repr.plot.height=6)
Checkpoint<-(ndf$Trial == 10)|(ndf$Trial == 20)|(ndf$Trial == 30)
COND<-Checkpoint&(ndf$SocInfo != 2)

ggplot(ndf[COND,], aes(x=Fitness, group = as.factor(SocInfo), color = as.factor(SocInfo), fill = as.factor(SocInfo))) +  
geom_density(aes(color = as.factor(SocInfo)), alpha = 0.4)+
facet_grid(Season ~ Trial, labeller = labeli_temp)+
scale_color_manual(values = CPalet, name = "Condition",labels = c("Asocial", "Negative","Positive"))+
scale_fill_manual(values = CPalet, name = "Condition",labels = c("Asocial", "Negative","Positive"))+
theme_bw()
```

#### positive v. asocial¶

In [15]:

```
options(repr.plot.width=12, repr.plot.height=6)
Checkpoint<-(ndf$Trial == 10)|(ndf$Trial == 20)|(ndf$Trial == 30)
COND<-Checkpoint&(ndf$SocInfo != 1)

ggplot(ndf[COND,], aes(x=Fitness, group = as.factor(SocInfo), color = as.factor(SocInfo), fill = as.factor(SocInfo))) +  
geom_histogram(position = "identity", alpha = 0.4, bins = 12)+
geom_density(aes(color = as.factor(SocInfo)))+
facet_grid(Season ~ Trial)+scale_color_manual(values = CPalet[c(1,3)], name = "Condition",labels = c("Asocial","Positive"))+
scale_fill_manual(values = CPalet[c(1,3)], name = "Condition",labels = c("Asocial","Positive"))+
theme_bw()
```

### Social Infomation¶

- extracting trials in which participants could use social infomation

In [16]:

```
SocL_index <-((ndf$Trial)%%3) ==  0
socndf<-ndf[SocL_index,]
```

#### Looking Time¶

In [17]:

```
labeli <- as_labeller(c("1" = "Season 1",
                         "2" = "Season 2",
                         "3" = "Season 3"))


options(repr.plot.width=5, repr.plot.height=4)
ggplot(socndf[socndf$Season!=1,], aes(x=Trial,y=AggLT, group=SocInfo, color = as.factor(SocInfo), fill= as.factor(SocInfo), shape = as.factor(SocInfo))) +
stat_summary(fun="mean", geom="line") +
stat_summary(fun="mean", geom="point") +
stat_summary(fun = mean,
               fun.min = function(x) mean(x) -1.96*sd(x)/sqrt(length(x)), 
               fun.max = function(x) mean(x) + 1.96*sd(x)/sqrt(length(x)), 
               geom = "pointrange") +
facet_grid(Season ~ ., labeller = labeli)+
scale_color_manual(values = CPalet, name = "Condition",labels = c("Asocial", "Negative","Positive"))+
scale_fill_manual(values = CPalet, name = "Condition",labels = c("Asocial", "Negative","Positive"))+
scale_shape_manual(values = c(21,25,24), name = "Condition",labels = c("Asocial", "Negative","Positive"))+

theme_bw()+
labs(y = "Total Looking Time")
```

#### Click Frequency¶

In [18]:

```
options(repr.plot.width=5, repr.plot.height=4)
ggplot(socndf[socndf$Season!=1,], aes(x=Trial,y=AggClick, group=SocInfo, color = as.factor(SocInfo), fill= as.factor(SocInfo), shape = as.factor(SocInfo))) +
stat_summary(fun="mean", geom="line") +
stat_summary(fun="mean", geom="point") +
stat_summary(fun = mean,
               fun.min = function(x) mean(x) -1.96*sd(x)/sqrt(length(x)), 
               fun.max = function(x) mean(x) + 1.96*sd(x)/sqrt(length(x)), 
               geom = "pointrange") +
facet_grid(Season ~ ., labeller = labeli)+
scale_color_manual(values = CPalet, name = "Condition",labels = c("Asocial", "Negative","Positive"))+
scale_fill_manual(values = CPalet, name = "Condition",labels = c("Asocial", "Negative","Positive"))+
scale_shape_manual(values = c(21,25,24), name = "Condition",labels = c("Asocial", "Negative","Positive"))+
theme_bw()+
labs(y = "Total Frequency of Click")
```

### Parameters Shift¶

#### Length¶

In [19]:

```
options(repr.plot.width=4.5, repr.plot.height=5.4)

Lhline.data <- data.frame(Season = 1:3, Length = c(30,36,39))


ggplot(ndf, aes(x=Trial,y=Length,
                group=SocInfo, color = as.factor(SocInfo), fill= as.factor(SocInfo), shape = as.factor(SocInfo))) +
stat_summary(fun="mean", geom="line") +
stat_summary(fun="mean", geom="point", size = 1.8) +
stat_summary(fun = mean,
               fun.min = function(x) mean(x) - sd(x)/sqrt(length(x)), 
               fun.max = function(x) mean(x) +sd(x)/sqrt(length(x)), 
               geom = "linerange",) +
geom_hline(aes(yintercept = Length), Lhline.data, linetype="dashed")+
facet_grid(Season ~ .,labeller = labeli)+
scale_color_manual(values = CPalet, name = "Condition",labels = c("Asocial", "Negative","Positive"))+
scale_fill_manual(values = CPalet, name = "Condition",labels = c("Asocial", "Negative","Positive"))+
scale_shape_manual(values = c(21,25,24), name = "Condition",labels = c("Asocial", "Negative","Positive"))+
theme_bw()
```

#### Width¶

In [20]:

```
options(repr.plot.width=4.5, repr.plot.height=5.4)
Whline.data <- data.frame(Season = 1:3, Width = c(63,4,17))


ggplot(ndf, aes(x=Trial,y=Width,
                group=SocInfo, color = as.factor(SocInfo), fill= as.factor(SocInfo), shape = as.factor(SocInfo))) +
stat_summary(fun="mean", geom="line") +
stat_summary(fun="mean", geom="point", size = 1.8) +
stat_summary(fun = mean,
               fun.min = function(x) mean(x) - sd(x)/sqrt(length(x)), 
               fun.max = function(x) mean(x) +sd(x)/sqrt(length(x)), 
               geom = "linerange") +
geom_hline(aes(yintercept = Width), Whline.data, linetype="dashed")+
facet_grid(Season ~ .,labeller = labeli)+
scale_color_manual(values = CPalet, name = "Condition",labels = c("Asocial", "Negative","Positive"))+
scale_fill_manual(values = CPalet, name = "Condition",labels = c("Asocial", "Negative","Positive"))+
scale_shape_manual(values = c(21,25,24), name = "Condition",labels = c("Asocial", "Negative","Positive"))+
theme_bw()
```

#### Thickness¶

In [21]:

```
options(repr.plot.width=4.5, repr.plot.height=5.4)
Thline.data <- data.frame(Season = 1:3, Thick = c(34,57,84))


ggplot(ndf, aes(x=Trial,y=Thick,
                group=SocInfo, color = as.factor(SocInfo), fill= as.factor(SocInfo), shape = as.factor(SocInfo))) +
stat_summary(fun="mean", geom="line") +
stat_summary(fun="mean", geom="point", size = 1.8) +
stat_summary(fun = mean,
               fun.min = function(x) mean(x) - sd(x)/sqrt(length(x)), 
               fun.max = function(x) mean(x) +sd(x)/sqrt(length(x)), 
               geom = "linerange") +
geom_hline(aes(yintercept = Thick), Thline.data, linetype="dashed")+
facet_grid(Season ~ .,labeller = labeli)+
scale_color_manual(values = CPalet, name = "Condition",labels = c("Asocial", "Negative","Positive"))+
scale_fill_manual(values = CPalet, name = "Condition",labels = c("Asocial", "Negative","Positive"))+
scale_shape_manual(values = c(21,25,24), name = "Condition",labels = c("Asocial", "Negative","Positive"))+
theme_bw()
```

## How participants have changed parameta?¶

### Preparation¶

Making data based on X(t) - X(t-1)

In [22]:

```
Lchange<- c(ndf$Length,0) - c(0, ndf $Length)
#Lchange<-Lchange[-1]
#Lchange [ndf$trial == 1]<- -9999
Lchange<-Lchange[-length(Lchange)]
IfLchange<- (Lchange != 0)+0

IfLchange[ndf$trial == 1]<- -9999

Wchange<- c(ndf$Width,0) - c(0,ndf $Width)
#Wchange<-Wchange[-1]
#Wchange[ndf$trial == 1]<- -9999
Wchange<-Wchange[-length(Wchange)]
IfWchange<- (Wchange != 0)+0

#IfWchange[ndf$trial == 1]<- -9999

Tchange<- c(ndf$Thick,0) - c(0,ndf $Thick)
#Tchange<-Tchange[-1]
#Tchange[ndf$trial == 1]<- -9999
Tchange<-Tchange[-length(Tchange)]
IfTchange<- (Tchange != 0)+0
#IfTchange[ndf$trial == 1]<- -9999


ndf<-mutate(ndf, Lchange,Wchange,Tchange,IfLchange,IfWchange , IfTchange)
```

In [23]:

```
ndf<-mutate(ndf, Lchange,Wchange,Tchange)
```

In [24]:

```
IfSchange<- c(ndf$Shape,0) != c(0,ndf $Shape)
#IfSchange[ndf$trial ==1]<- -999
IfSchange<-IfSchange[-length(IfSchange)]
#IfSchange<-IfSchange[-1]
#IfSchange

IfCchange<- c(ndf$Color,0) != c(0,ndf$Color)
#IfCchange[ndf$trial == 1]<- -999
IfCchange<-IfCchange[-length(IfCchange)]
#IfCchange<-IfCchange[-1]
#fCchange

ndf<-mutate(ndf, IfSchange , IfCchange)
```

### Number of changes in dimention for each trial¶

In [25]:

```
SumChange<-ndf$IfLchange + ndf$IfWchange + ndf$IfTchange + ndf$IfSchange + ndf$IfCchange
ndf<-mutate(ndf, SumChange)
```

In [26]:

```
SumChangeCont<-ndf$IfLchange + ndf$IfWchange + ndf$IfTchange 
ndf<-mutate(ndf, SumChangeCont)
```

In [27]:

```
options(repr.plot.width=6, repr.plot.height=7)
ggplot(ndf[ndf$Trial != 1,], aes(x=Trial,y=SumChange, 
                                , group=SocInfo, colour = as.factor(SocInfo),  shape=as.factor(SocInfo), fill = as.factor(SocInfo))) +
stat_summary(fun="mean", geom="line") +
stat_summary(fun="mean", geom="point") +
stat_summary(fun = mean,
               fun.min = function(x) mean(x) - sd(x)/sqrt(length(x)), 
               fun.max = function(x) mean(x) + sd(x)/sqrt(length(x)), 
               geom = "pointrange") +
facet_grid(Season ~ . ,labeller = labeli) +
scale_color_manual(values = CPalet, name = "Condition",labels = c("Asocial", "Negative","Positive"))+
scale_fill_manual(values = CPalet, name = "Condition",labels = c("Asocial", "Negative","Positive"))+
scale_shape_manual(values = c(21,25,24), name = "Condition",labels = c("Asocial", "Negative","Positive"))+theme_bw()
```

In [28]:

```
options(repr.plot.width=6, repr.plot.height=7)
ggplot(ndf[ndf$Trial != 1,], aes(x=Trial,y=SumChangeCont, 
                                , group=SocInfo, colour = as.factor(SocInfo),  shape=as.factor(SocInfo), fill = as.factor(SocInfo))) +
stat_summary(fun="mean", geom="line") +
stat_summary(fun="mean", geom="point") +
stat_summary(fun = mean,
               fun.min = function(x) mean(x) - sd(x)/sqrt(length(x)), 
               fun.max = function(x) mean(x) + sd(x)/sqrt(length(x)), 
               geom = "pointrange") +
facet_grid(Season ~ . ,labeller = labeli) +
scale_color_manual(values = CPalet, name = "Condition",labels = c("Asocial", "Negative","Positive"))+
scale_fill_manual(values = CPalet, name = "Condition",labels = c("Asocial", "Negative","Positive"))+
scale_shape_manual(values = c(21,25,24), name = "Condition",labels = c("Asocial", "Negative","Positive"))+theme_bw()
```

#### Mode and Median¶

In [29]:

```
table(ndf[ndf$Trial != 1,]$SumChange)
barplot(table(ndf[ndf$Trial != 1,]$SumChange))
```

```
   0    1    2    3    4    5 
1379 5180 2173 1205  371  132
```

In [30]:

```
table(ndf[ndf$Trial != 1,]$SumChangeCont)
barplot(table(ndf[ndf$Trial != 1,]$SumChangeCont))
```

```
   0    1    2    3 
3151 4213 1776 1300
```

## t-test¶

### To calculate Block Mean¶

Here, first transform the tidydata into spread.
Then calulate the mean of block.
Then return to tidy data (which is not necessary)

In [31]:

```
aimdata<-ndf[,c('ID','Season','SocInfo','Trial','Fitness')]

sprd<- tidyr::spread(aimdata, key = Trial, value = Fitness)
```

#### Calculate Overall Performance¶

In [32]:

```
head(sprd)
colnames(sprd) 


#Forcenum to identify trial num and those not
ForceNum<-as.numeric(colnames(sprd) )
ForceNum[is.na(ForceNum)]<-0
ForceNum

DecisionTrialsIndex<- ForceNum  >0
DecisionTrialsIndex
```

A data.frame: 6 × 33

|  | ID | Season | SocInfo | 1 | 2 | 3 | 4 | 5 | 6 | 7 | ⋯ | 21 | 22 | 23 | 24 | 25 | 26 | 27 | 28 | 29 | 30 |
| --- | --- | --- | --- | --- | --- | --- | --- | --- | --- | --- | --- | --- | --- | --- | --- | --- | --- | --- | --- | --- | --- |
|  | <int> | <int> | <int> | <int> | <int> | <int> | <int> | <int> | <int> | <int> | ⋯ | <int> | <int> | <int> | <int> | <int> | <int> | <int> | <int> | <int> | <int> |
| 1 | 1 | 1 | 0 | 274 | 118 | 334 | 407 | 441 | 307 | 348 | ⋯ | 594 | 611 | 619 | 568 | 587 | 570 | 507 | 568 | 532 | 585 |
| 2 | 1 | 2 | 0 | 363 | 226 | 608 | 532 | 552 | 589 | 548 | ⋯ | 753 | 747 | 747 | 742 | 739 | 750 | 752 | 751 | 751 | 751 |
| 3 | 1 | 3 | 0 | 238 | 261 | 355 | 346 | 367 | 409 | 325 | ⋯ | 845 | 849 | 850 | 847 | 874 | 892 | 908 | 938 | 956 | 973 |
| 4 | 2 | 1 | 0 | 274 | 274 | 233 | 233 | 143 | 333 | 369 | ⋯ | 527 | 527 | 527 | 527 | 527 | 527 | 527 | 527 | 598 | 598 |
| 5 | 2 | 2 | 0 | 363 | 363 | 406 | 428 | 469 | 611 | 530 | ⋯ | 613 | 676 | 676 | 673 | 647 | 673 | 686 | 701 | 718 | 718 |
| 6 | 2 | 3 | 0 | 238 | 244 | 453 | 440 | 440 | 324 | 533 | ⋯ | 652 | 654 | 656 | 661 | 661 | 656 | 656 | 644 | 614 | 656 |

1. 'ID'
2. 'Season'
3. 'SocInfo'
4. '1'
5. '2'
6. '3'
7. '4'
8. '5'
9. '6'
10. '7'
11. '8'
12. '9'
13. '10'
14. '11'
15. '12'
16. '13'
17. '14'
18. '15'
19. '16'
20. '17'
21. '18'
22. '19'
23. '20'
24. '21'
25. '22'
26. '23'
27. '24'
28. '25'
29. '26'
30. '27'
31. '28'
32. '29'
33. '30'

```
Warning message in eval(expr, envir, enclos):
“ 強制変換により NA が生成されました ”
```

1. 0
2. 0
3. 0
4. 1
5. 2
6. 3
7. 4
8. 5
9. 6
10. 7
11. 8
12. 9
13. 10
14. 11
15. 12
16. 13
17. 14
18. 15
19. 16
20. 17
21. 18
22. 19
23. 20
24. 21
25. 22
26. 23
27. 24
28. 25
29. 26
30. 27
31. 28
32. 29
33. 30

1. FALSE
2. FALSE
3. FALSE
4. TRUE
5. TRUE
6. TRUE
7. TRUE
8. TRUE
9. TRUE
10. TRUE
11. TRUE
12. TRUE
13. TRUE
14. TRUE
15. TRUE
16. TRUE
17. TRUE
18. TRUE
19. TRUE
20. TRUE
21. TRUE
22. TRUE
23. TRUE
24. TRUE
25. TRUE
26. TRUE
27. TRUE
28. TRUE
29. TRUE
30. TRUE
31. TRUE
32. TRUE
33. TRUE

In [33]:

```
head(sprd[DecisionTrialsIndex])
SumScore<-apply(sprd[DecisionTrialsIndex],1,mean)
sprd2<-mutate(sprd, SumScore)
```

A data.frame: 6 × 30

|  | 1 | 2 | 3 | 4 | 5 | 6 | 7 | 8 | 9 | 10 | ⋯ | 21 | 22 | 23 | 24 | 25 | 26 | 27 | 28 | 29 | 30 |
| --- | --- | --- | --- | --- | --- | --- | --- | --- | --- | --- | --- | --- | --- | --- | --- | --- | --- | --- | --- | --- | --- |
|  | <int> | <int> | <int> | <int> | <int> | <int> | <int> | <int> | <int> | <int> | ⋯ | <int> | <int> | <int> | <int> | <int> | <int> | <int> | <int> | <int> | <int> |
| 1 | 274 | 118 | 334 | 407 | 441 | 307 | 348 | 442 | 412 | 470 | ⋯ | 594 | 611 | 619 | 568 | 587 | 570 | 507 | 568 | 532 | 585 |
| 2 | 363 | 226 | 608 | 532 | 552 | 589 | 548 | 589 | 589 | 589 | ⋯ | 753 | 747 | 747 | 742 | 739 | 750 | 752 | 751 | 751 | 751 |
| 3 | 238 | 261 | 355 | 346 | 367 | 409 | 325 | 476 | 349 | 459 | ⋯ | 845 | 849 | 850 | 847 | 874 | 892 | 908 | 938 | 956 | 973 |
| 4 | 274 | 274 | 233 | 233 | 143 | 333 | 369 | 409 | 386 | 395 | ⋯ | 527 | 527 | 527 | 527 | 527 | 527 | 527 | 527 | 598 | 598 |
| 5 | 363 | 363 | 406 | 428 | 469 | 611 | 530 | 638 | 556 | 678 | ⋯ | 613 | 676 | 676 | 673 | 647 | 673 | 686 | 701 | 718 | 718 |
| 6 | 238 | 244 | 453 | 440 | 440 | 324 | 533 | 533 | 563 | 492 | ⋯ | 652 | 654 | 656 | 661 | 661 | 656 | 656 | 644 | 614 | 656 |

#### Making into tidy data¶

In [34]:

```
S<-1
print(S)
Ind<-(sprd2$Season ==S)&(sprd2$SocInfo ==0)
Neg<-(sprd2$Season ==S)&(sprd2$SocInfo ==1)
Pos<-(sprd2$Season ==S)&(sprd2$SocInfo ==2)
print("Neg")
describe(sprd2[Neg,]$SumScore)

effsize::cohen.d( sprd2[Neg,]$SumScore,  sprd2[Ind,]$SumScore)
t.test( sprd2[Neg,]$SumScore,  sprd2[Ind,]$SumScore)

print("Pos")
describe(sprd2[Pos,]$SumScore)
effsize::cohen.d(sprd2[Pos,]$SumScore,  sprd2[Ind,]$SumScore)
t.test( sprd2[Pos,]$SumScore,  sprd2[Ind,]$SumScore)
sd(sprd2[Pos,]$SumScore)
   
print("Ind")   
describe(sprd2[Ind,]$SumScore)
```

```
[1] 1
[1] "Neg"
```

A psych: 1 × 13

|  | vars | n | mean | sd | median | trimmed | mad | min | max | range | skew | kurtosis | se |
| --- | --- | --- | --- | --- | --- | --- | --- | --- | --- | --- | --- | --- | --- |
|  | <dbl> | <dbl> | <dbl> | <dbl> | <dbl> | <dbl> | <dbl> | <dbl> | <dbl> | <dbl> | <dbl> | <dbl> | <dbl> |
| X1 | 1 | 40 | 613.3358 | 162.884 | 629.1 | 619.0635 | 180.9019 | 245.9667 | 876.0667 | 630.1 | -0.3074469 | -0.8106113 | 25.75422 |

```
Cohen's d

d estimate: 0.4875573 (small)
95 percent confidence interval:
     lower      upper 
0.03582489 0.93928964
```

```
	Welch Two Sample t-test

data:  sprd2[Neg, ]$SumScore and sprd2[Ind, ]$SumScore
t = 2.1804, df = 71.964, p-value = 0.0325
alternative hypothesis: true difference in means is not equal to 0
95 percent confidence interval:
   5.995694 133.867639
sample estimates:
mean of x mean of y 
 613.3358  543.4042
```

```
[1] "Pos"
```

A psych: 1 × 13

|  | vars | n | mean | sd | median | trimmed | mad | min | max | range | skew | kurtosis | se |
| --- | --- | --- | --- | --- | --- | --- | --- | --- | --- | --- | --- | --- | --- |
|  | <dbl> | <dbl> | <dbl> | <dbl> | <dbl> | <dbl> | <dbl> | <dbl> | <dbl> | <dbl> | <dbl> | <dbl> | <dbl> |
| X1 | 1 | 40 | 599.9033 | 126.1546 | 618.7833 | 602.7167 | 135.8803 | 286.6333 | 830.6 | 543.9667 | -0.2829051 | -0.6114818 | 19.94679 |

```
Cohen's d

d estimate: 0.4572944 (small)
95 percent confidence interval:
      lower       upper 
0.006346724 0.908242095
```

```
	Welch Two Sample t-test

data:  sprd2[Pos, ]$SumScore and sprd2[Ind, ]$SumScore
t = 2.0451, df = 77.859, p-value = 0.04423
alternative hypothesis: true difference in means is not equal to 0
95 percent confidence interval:
   1.496789 111.501544
sample estimates:
mean of x mean of y 
 599.9033  543.4042
```

126.154589922066

```
[1] "Ind"
```

A psych: 1 × 13

|  | vars | n | mean | sd | median | trimmed | mad | min | max | range | skew | kurtosis | se |
| --- | --- | --- | --- | --- | --- | --- | --- | --- | --- | --- | --- | --- | --- |
|  | <dbl> | <dbl> | <dbl> | <dbl> | <dbl> | <dbl> | <dbl> | <dbl> | <dbl> | <dbl> | <dbl> | <dbl> | <dbl> |
| X1 | 1 | 40 | 543.4042 | 120.8913 | 546.4167 | 544.4188 | 113.839 | 272.0333 | 796.4 | 524.3667 | -0.05513352 | -0.2808927 | 19.11459 |

In [35]:

```
S<-2
print(S)
Ind<-(sprd2$Season ==S)&(sprd2$SocInfo ==0)
Neg<-(sprd2$Season ==S)&(sprd2$SocInfo ==1)
Pos<-(sprd2$Season ==S)&(sprd2$SocInfo ==2)
print("Neg")
describe(sprd2[Neg,]$SumScore)

effsize::cohen.d( sprd2[Neg,]$SumScore,  sprd2[Ind,]$SumScore)
t.test( sprd2[Neg,]$SumScore,  sprd2[Ind,]$SumScore)

print("Pos")
describe(sprd2[Pos,]$SumScore)
effsize::cohen.d(sprd2[Pos,]$SumScore,  sprd2[Ind,]$SumScore)
t.test( sprd2[Pos,]$SumScore,  sprd2[Ind,]$SumScore)
sd(sprd2[Pos,]$SumScore)
   
print("Ind")   
describe(sprd2[Ind,]$SumScore)
```

```
[1] 2
[1] "Neg"
```

A psych: 1 × 13

|  | vars | n | mean | sd | median | trimmed | mad | min | max | range | skew | kurtosis | se |
| --- | --- | --- | --- | --- | --- | --- | --- | --- | --- | --- | --- | --- | --- |
|  | <dbl> | <dbl> | <dbl> | <dbl> | <dbl> | <dbl> | <dbl> | <dbl> | <dbl> | <dbl> | <dbl> | <dbl> | <dbl> |
| X1 | 1 | 40 | 638.1708 | 111.2615 | 650.6333 | 641.1073 | 78.28128 | 379.2333 | 868.8333 | 489.6 | -0.2411127 | -0.009246913 | 17.59199 |

```
Cohen's d

d estimate: 0.1175431 (negligible)
95 percent confidence interval:
     lower      upper 
-0.3280081  0.5630943
```

```
	Welch Two Sample t-test

data:  sprd2[Neg, ]$SumScore and sprd2[Ind, ]$SumScore
t = 0.52567, df = 76.666, p-value = 0.6006
alternative hypothesis: true difference in means is not equal to 0
95 percent confidence interval:
 -34.27521  58.86021
sample estimates:
mean of x mean of y 
 638.1708  625.8783
```

```
[1] "Pos"
```

A psych: 1 × 13

|  | vars | n | mean | sd | median | trimmed | mad | min | max | range | skew | kurtosis | se |
| --- | --- | --- | --- | --- | --- | --- | --- | --- | --- | --- | --- | --- | --- |
|  | <dbl> | <dbl> | <dbl> | <dbl> | <dbl> | <dbl> | <dbl> | <dbl> | <dbl> | <dbl> | <dbl> | <dbl> | <dbl> |
| X1 | 1 | 40 | 822.3208 | 86.98316 | 842.5667 | 833.2458 | 64.4931 | 531.4333 | 939.7 | 408.2667 | -1.275 | 1.797705 | 13.75325 |

```
Cohen's d

d estimate: 2.126947 (large)
95 percent confidence interval:
   lower    upper 
1.569957 2.683938
```

```
	Welch Two Sample t-test

data:  sprd2[Pos, ]$SumScore and sprd2[Ind, ]$SumScore
t = 9.512, df = 77.016, p-value = 1.239e-14
alternative hypothesis: true difference in means is not equal to 0
95 percent confidence interval:
 155.3191 237.5659
sample estimates:
mean of x mean of y 
 822.3208  625.8783
```

86.9831632814331

```
[1] "Ind"
```

A psych: 1 × 13

|  | vars | n | mean | sd | median | trimmed | mad | min | max | range | skew | kurtosis | se |
| --- | --- | --- | --- | --- | --- | --- | --- | --- | --- | --- | --- | --- | --- |
|  | <dbl> | <dbl> | <dbl> | <dbl> | <dbl> | <dbl> | <dbl> | <dbl> | <dbl> | <dbl> | <dbl> | <dbl> | <dbl> |
| X1 | 1 | 40 | 625.8783 | 97.43848 | 613.65 | 627.4521 | 104.8692 | 409.5 | 845.1667 | 435.6667 | 0.02798789 | -0.4484114 | 15.40638 |

In [36]:

```
S<-3
print(S)
Ind<-(sprd2$Season ==S)&(sprd2$SocInfo ==0)
Neg<-(sprd2$Season ==S)&(sprd2$SocInfo ==1)
Pos<-(sprd2$Season ==S)&(sprd2$SocInfo ==2)
print("Neg")
describe(sprd2[Neg,]$SumScore)

effsize::cohen.d( sprd2[Neg,]$SumScore,  sprd2[Ind,]$SumScore)
t.test( sprd2[Neg,]$SumScore,  sprd2[Ind,]$SumScore)

print("Pos")
describe(sprd2[Pos,]$SumScore)
effsize::cohen.d(sprd2[Pos,]$SumScore,  sprd2[Ind,]$SumScore)
t.test( sprd2[Pos,]$SumScore,  sprd2[Ind,]$SumScore)
sd(sprd2[Pos,]$SumScore)
   
print("Ind")   
describe(sprd2[Ind,]$SumScore)
```

```
[1] 3
[1] "Neg"
```

A psych: 1 × 13

|  | vars | n | mean | sd | median | trimmed | mad | min | max | range | skew | kurtosis | se |
| --- | --- | --- | --- | --- | --- | --- | --- | --- | --- | --- | --- | --- | --- |
|  | <dbl> | <dbl> | <dbl> | <dbl> | <dbl> | <dbl> | <dbl> | <dbl> | <dbl> | <dbl> | <dbl> | <dbl> | <dbl> |
| X1 | 1 | 40 | 626.9225 | 176.381 | 629.9167 | 632.301 | 195.4561 | 248.1 | 935.0667 | 686.9667 | -0.1771385 | -0.8564523 | 27.88828 |

```
Cohen's d

d estimate: -0.07461483 (negligible)
95 percent confidence interval:
     lower      upper 
-0.5199366  0.3707070
```

```
	Welch Two Sample t-test

data:  sprd2[Neg, ]$SumScore and sprd2[Ind, ]$SumScore
t = -0.33369, df = 75.512, p-value = 0.7395
alternative hypothesis: true difference in means is not equal to 0
95 percent confidence interval:
 -84.38077  60.16577
sample estimates:
mean of x mean of y 
 626.9225  639.0300
```

```
[1] "Pos"
```

A psych: 1 × 13

|  | vars | n | mean | sd | median | trimmed | mad | min | max | range | skew | kurtosis | se |
| --- | --- | --- | --- | --- | --- | --- | --- | --- | --- | --- | --- | --- | --- |
|  | <dbl> | <dbl> | <dbl> | <dbl> | <dbl> | <dbl> | <dbl> | <dbl> | <dbl> | <dbl> | <dbl> | <dbl> | <dbl> |
| X1 | 1 | 40 | 796.9467 | 116.2483 | 832.8667 | 807.8573 | 97.55508 | 424.2333 | 945.7333 | 521.5 | -1.019951 | 0.6190375 | 18.38047 |

```
Cohen's d

d estimate: 1.192642 (large)
95 percent confidence interval:
    lower     upper 
0.7095176 1.6757655
```

```
	Welch Two Sample t-test

data:  sprd2[Pos, ]$SumScore and sprd2[Ind, ]$SumScore
t = 5.3337, df = 74.107, p-value = 1.005e-06
alternative hypothesis: true difference in means is not equal to 0
95 percent confidence interval:
  98.92367 216.90966
sample estimates:
mean of x mean of y 
 796.9467  639.0300
```

116.248317035883

```
[1] "Ind"
```

A psych: 1 × 13

|  | vars | n | mean | sd | median | trimmed | mad | min | max | range | skew | kurtosis | se |
| --- | --- | --- | --- | --- | --- | --- | --- | --- | --- | --- | --- | --- | --- |
|  | <dbl> | <dbl> | <dbl> | <dbl> | <dbl> | <dbl> | <dbl> | <dbl> | <dbl> | <dbl> | <dbl> | <dbl> | <dbl> |
| X1 | 1 | 40 | 639.03 | 146.8016 | 640.8667 | 646.3583 | 180.3336 | 264.2667 | 852.4333 | 588.1667 | -0.409219 | -0.7917324 | 23.21137 |

## F-test (equal variance)¶

Comparing the varience of the final trial (Trial == 30)

### season 1¶

In [37]:

```
S = 1

AsocLast <- ndf[(ndf$SocInfo == 0)&(ndf$Trial == 30)&(ndf$Season == S),]
NegLast<-ndf[(ndf$SocInfo == 1)&(ndf$Trial == 30)&(ndf$Season == S),]
PosLast<-ndf[(ndf$SocInfo == 2)&(ndf$Trial == 30)&(ndf$Season == S),]

var.test(AsocLast$Fitness, NegLast$Fitness)
var.test(AsocLast$Fitness, PosLast$Fitness)
```

```
	F test to compare two variances

data:  AsocLast$Fitness and NegLast$Fitness
F = 0.89402, num df = 39, denom df = 39, p-value = 0.7282
alternative hypothesis: true ratio of variances is not equal to 1
95 percent confidence interval:
 0.4728489 1.6903488
sample estimates:
ratio of variances 
         0.8940244
```

```
	F test to compare two variances

data:  AsocLast$Fitness and PosLast$Fitness
F = 1.1717, num df = 39, denom df = 39, p-value = 0.6233
alternative hypothesis: true ratio of variances is not equal to 1
95 percent confidence interval:
 0.6196867 2.2152674
sample estimates:
ratio of variances 
          1.171653
```

### season 2¶

In [38]:

```
S = 2

AsocLast <- ndf[(ndf$SocInfo == 0)&(ndf$Trial == 30)&(ndf$Season == S),]
NegLast<-ndf[(ndf$SocInfo == 1)&(ndf$Trial == 30)&(ndf$Season == S),]
PosLast<-ndf[(ndf$SocInfo == 2)&(ndf$Trial == 30)&(ndf$Season == S),]

var.test(AsocLast$Fitness, NegLast$Fitness)
var.test(AsocLast$Fitness, PosLast$Fitness)
```

```
	F test to compare two variances

data:  AsocLast$Fitness and NegLast$Fitness
F = 0.71164, num df = 39, denom df = 39, p-value = 0.2924
alternative hypothesis: true ratio of variances is not equal to 1
95 percent confidence interval:
 0.3763838 1.3455037
sample estimates:
ratio of variances 
          0.711636
```

```
	F test to compare two variances

data:  AsocLast$Fitness and PosLast$Fitness
F = 2.1345, num df = 39, denom df = 39, p-value = 0.02005
alternative hypothesis: true ratio of variances is not equal to 1
95 percent confidence interval:
 1.128957 4.035814
sample estimates:
ratio of variances 
           2.13454
```

### season 3¶

In [39]:

```
S = 3

AsocLast <- ndf[(ndf$SocInfo == 0)&(ndf$Trial == 30)&(ndf$Season == S),]
NegLast<-ndf[(ndf$SocInfo == 1)&(ndf$Trial == 30)&(ndf$Season == S),]
PosLast<-ndf[(ndf$SocInfo == 2)&(ndf$Trial == 30)&(ndf$Season == S),]

var.test(AsocLast$Fitness, NegLast$Fitness)
var.test(AsocLast$Fitness, PosLast$Fitness)
```

```
	F test to compare two variances

data:  AsocLast$Fitness and NegLast$Fitness
F = 0.85957, num df = 39, denom df = 39, p-value = 0.6389
alternative hypothesis: true ratio of variances is not equal to 1
95 percent confidence interval:
 0.4546245 1.6252001
sample estimates:
ratio of variances 
         0.8595672
```

```
	F test to compare two variances

data:  AsocLast$Fitness and PosLast$Fitness
F = 5.9827, num df = 39, denom df = 39, p-value = 1.634e-07
alternative hypothesis: true ratio of variances is not equal to 1
95 percent confidence interval:
  3.164256 11.311639
sample estimates:
ratio of variances 
          5.982718
```
